# Proanthocyanidins and Where to Find Them: A Meta-Analytic Approach to Investigate Their Chemistry, Biosynthesis, Distribution, and Effect on Human Health

**DOI:** 10.3390/antiox10081229

**Published:** 2021-07-30

**Authors:** Giuseppe Mannino, Giorgia Chinigò, Graziella Serio, Tullio Genova, Carla Gentile, Luca Munaron, Cinzia Margherita Bertea

**Affiliations:** 1 Plant Physiology Unit, Department of Life Sciences and Systems Biology, University of Turin, Via Quarello 15/A, 10135 Turin, Italy; giuseppe.mannino@unito.it (G.M.); cinzia.bertea@unito.it (C.M.B.); 2 Department of Life Sciences and Systems Biology, University of Torino, Via Accademia Albertina 13, 10123 Turin, Italy; giorgia.chinigo@unito.it (G.C.); tullio.genova@unito.it (T.G.); luca.munaron@unito.it (L.M.); 3 Department of Biological, Chemical and Pharmaceutical Sciences and Technologies (STEBICEF), University of Palermo, Viale delle Scienze, 90128 Palermo, Italy; graziella.serio01@unipa.it

**Keywords:** polyphenols, proanthocyanidins, cluster analysis, forest plot, ecology, hyperglycemia, hyperlipidemia, cholesterol, inflammation, metabolic disorders

## Abstract

Proanthocyanidins (PACs) are a class of polyphenolic compounds that are attracting considerable interest in the nutraceutical field due to their potential health benefits. However, knowledge about the chemistry, biosynthesis, and distribution of PACs is limited. This review summarizes the main chemical characteristics and biosynthetic pathways and the main analytical methods aimed at their identification and quantification in raw plant matrices. Furthermore, meta-analytic approaches were used to identify the main plant sources in which PACs were contained and to investigate their potential effect on human health. In particular, a cluster analysis identified PACs in 35 different plant families and 60 different plant parts normally consumed in the human diet. On the other hand, a literature search, coupled with forest plot analyses, highlighted how PACs can be actively involved in both local and systemic effects. Finally, the potential mechanisms of action through which PACs may impact human health were investigated, focusing on their systemic hypoglycemic and lipid-lowering effects and their local anti-inflammatory actions on the intestinal epithelium. Overall, this review may be considered a complete report in which chemical, biosynthetic, ecological, and pharmacological aspects of PACs are discussed.

## 1. Introduction

It has repeatedly been shown that diet and physical/psychological well-being are closely linked. Moreover, the World Health Organization (WHO) states that nutrition and health are two fundamental and interconnected human rights [1]. Having a healthy and balanced diet not only provides energy and essential nutrients for growth and sustenance, but it is the main factor that actively influences and improves individual health status [2]. In this context, scientific evidence has shown that the intake of plant foods is closely related to the decrease of “civilization disease” incidences, such as neoplasms, diabetes, and several forms of dyslipidemia [3,4,5]. The potential beneficial effects derived from plant food consumption against these pathologies is related to the nutritional aspects of food as well as to the phytochemical profiles. Indeed, plants are the main sources of biologically active compounds that may exert a wide range of potential pharmacological activities [6,7].

The bioactive phytocomponents constitute a heterogeneous family of compounds, mainly without a nutritional role, but with high beneficial effects on human health. Although characterized by a high structural diversity, all of the phytochemical compounds peculiar to the plant kingdom generally have a low molecular weight, and as products of the plant secondary metabolism, they are not essential for the survival of the producer organism. Plants biosynthesize secondary metabolites to fulfil some physiological needs, such as the defense against herbivorous predators, pathogens, or insects, an interspecific competition with other plants, or to facilitate reproductive processes [8].

Although many plant bioactive compounds have been extensively investigated in the past, and are almost fully understood, others are still partially unknown from the functional point of view. In particular, proanthocyanidins (PACs) are still under investigation because many aspects related to their chemistry, biosynthesis, distribution, and role in plants and animals is still unknown. In particular, despite the fact that PACs derive from the condensation of at least two flavan-3-ol subunits, it is still unclear how the qualitative selection of the single monomers occurs. Moreover, to date, the mechanisms and reasons why plants preferably condense two flavan-3-ols through a single (B-type) or a double (B-type) bond, to form PACs, are not completely elucidated. From a biosynthetic point of view, although the pathways for PAC production is partly superimposable to that of other polyphenols, some aspects still remain only hypothesized, including the process of polymerization as well as the transport in plant vacuole. In addition, a detailed distribution of PACs in the plant kingdom has not been reported in the literature to date. Finally, many potential bioactivities of PACs in both plants and animals remain unknown, and those already investigated have been exclusively associated with their active redox properties [9,10]. However, not all of the demonstrated pharmacological activities can be explained through this action mechanism.

The aims of this review are to (i) elucidate the chemical structure of PACs; (ii) describe the biosynthetic pathways of PACs, highlighting what is currently unknown; (iii) use a meta-analytic approach to study the distribution of PACs in the plant kingdom; (iv) describe the ecological and physiological role of PACs in plants; (v) investigate the potential implications for human health due to the consumption of foods rich in PACs; (vi) use a meta-analytic approach to investigate a new potential action of PACs as glucose and cholesterol lowering agents.

## 2. Chemistry

**Figure 1:**
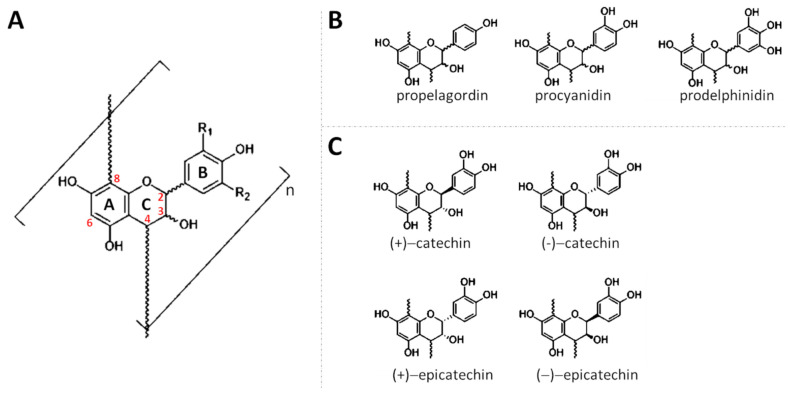
Chemical structures of some PAC monomers. Panel (**A**) shows the general chemical structure of flavan-3-ol monomers; Panel (**B**) shows a monomer of propelagordin, procyanidin, and prodelphinidin, which differ from each other in regard to the number and/or position of hydroxyl groups on the B-ring; Panel (**C**) shows the chemical structure of catechin and epicatechin, along with the respective enantiomers, which are different from each other in regard to the stereochemistry of flavonol heterocycle.

From a chemical point of view, PACs are oligomers or polymers resulting from the condensation of two or more 2-phenyl-3,4-dihydro-2H-chromen-3-ol (flavan-3-ol) units, which are composed of two aromatic benzyl rings joined by three carbon atoms that form an oxygenated heterocyclic ring (C6-C3-C6) (Figure 1A). In this context, oligomers derive from the condensation of 2–5 flavan-3-ol units, meanwhile polymers are much larger and derived by the condensation of 6–60 units [11,12,13]. Unlike other flavonoids, flavan-3-ols and, consequently PACs, have saturated A-ring and, hence, are non-planar molecules [14].

**Figure 2:**
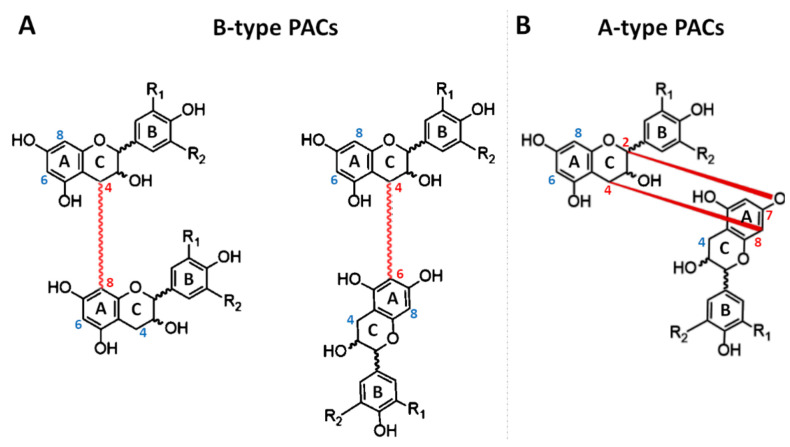
Linkage (red) that allows the polymerization of two monomers of flavan-3-ols, leading to the formation of B-type (**A**) or A-type (**B**) PACs.

Structurally, PACs may differ from each other in regard to (i) the number and position of hydroxyl groups linked to the aromatic rings (or B-ring); (ii) the stereochemistry of flavonol heterocycle (or C-ring); (iii) the type of linkage among the different units. According to the number and position of hydroxyl groups, three main PACs may be identified: propelagordins (having a hydroxyl group alone); procyanidins (having two hydroxyl groups), and prodelphinidins (having three hydroxyl groups) (Figure 1B). The reason why these molecules have these names is due to the fact that, in an acid environment, they may be hydrolyzed, following the formation of the corresponding anthocyanins [15]. The generated compounds, differently from the original oligomers or polymers, have a characteristic color ranging between red and blue [15]. Concerning the flavan-3-ol ring, since three carbons (C_2_, C_3_, and C_4_) of the heterocycle are asymmetric, they may present different configurations. At the beginning, it was assumed that the C_2_ configuration could be exclusively in R. However, although this configuration is certainly the most common in nature, other PACs composed of flavan-3-ols having C_2_ in S, have been detected [16,17]. When C_2_ presents a configuration in R, the prefix “ent-” is added to the nomenclature of the molecules. Additionally, the stereochemistry of C_3_ may exist both as 3R or 3S. Consequently, four different conformations are possible (2R,3R, 2R,3S, 2S,3R, and 2S,3S). When C_2_ and C_3_ have substituents spatially oriented in the same direction (2R, 3R or 2S,3S), the suffix “-epi” is added to the nomenclature. Moreover, because the orientation of C_3_ influences the optical rotation, the suffix “(+)”is assigned to flavan-3-ols with 3S configuration, meanwhile “(−)” is assigned to those having 3R configuration. Consequently, the (+)-epi-flavan-3-ols, (−)-epi-flavan-3-ols, (+)-flavan-3-ols, (−)-flavan-3-ols of a same molecule can exist (Figure 1C). Finally, the bond at C_4_ is always trans, with respect to the hydroxy group at C_3_ and, fortunately, this does not further complicate the nomenclature of these molecules [11,18]. The most famous example of this condition is represented by the catechin, which has been detected and identified in all four different forms (Figure 1C). In order to form a PAC, each flavan-3-ol unit is linked to another unit through C–C and/or C–O bonds [12,17,19]. The most usual linkages are C–C bonds established between the C_4_ of one flavan-3-ol unit and C_8_ or C_6_ of another (Figure 2A).

**Figure 3:**
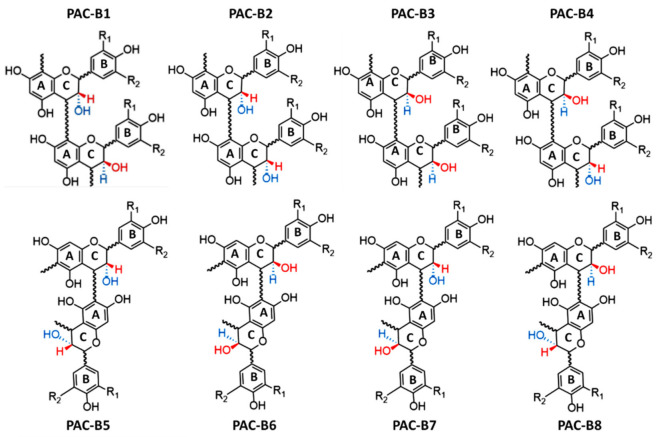
Chemical structures of the different B-type PACs, depending on the stereochemistry of substituents. The blue dashed line represents bonds that "sink" below the plane of the sheet, while the red wedged line indicates a chemical bond that is directed towards the observer.

**Figure 4:**
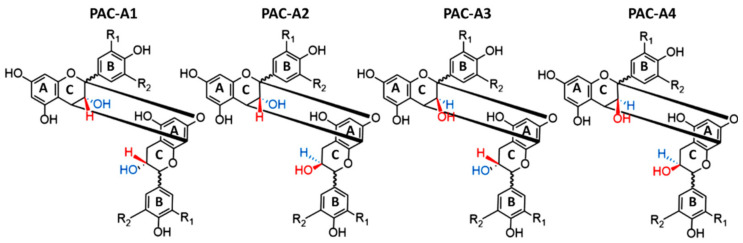
Chemical structures of the different A-type PACs depending on the stereochemistry of substituents. The blue dashed line represents bonds that "sink" below the plane of the sheet, while the red wedged line indicates a chemical bond that is directed towards the observer.

In this case, proanthocyanidins take the name of B-type. However, when the linkage between two units occurs, the hydroxyl group linked to the C-ring of each flavan-3-ol can be in either S or in R. Consequently, four different B-type PACs can be formed from C_4_–C_8_ linkages (B1–B4), and another four from C_4_–C_6_ (B1–B8) (Figure 3). Moreover, C–O bounds between O_7_ of one flavan-3-ol unit and C_2_ of another one can be established [20]. In this case, the PAC is named A-type (Figure 2B). For the same reasons previously described, in this case, four typologies of A-type PACs can be formed (Figure 4).

## 3. Biosynthesis, Transport, and Polymerization

### 3.1. Biosynthesis of Proanthocyanidins

**Figure 5:**
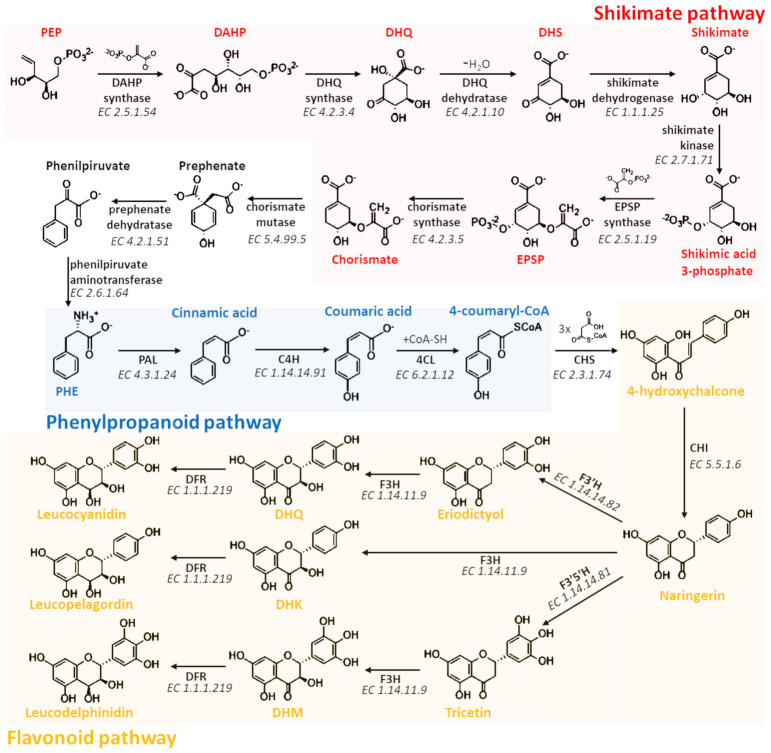
Biosynthetic pathway involved in the synthesis of leucoanthocyanidins, the key precursor compounds of flavan-3-ols. The pathway involves the shikimate (Red), phenylpropanoid (Blue), and flavonoid (Yellow) pathways.

**Figure 6:**
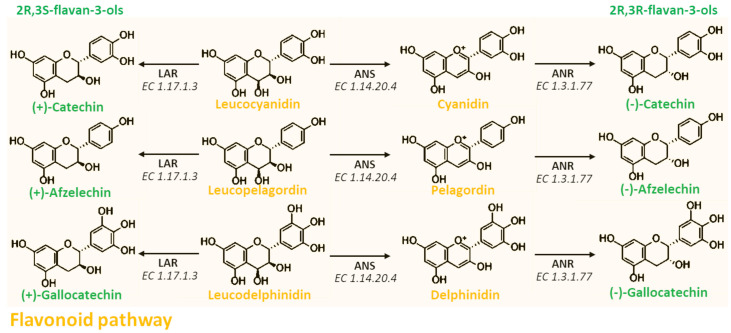
Biosynthetic reactions leading to the formation of 2R,3S-flavan3-ols or 2R,3R-flavan3-ols from the respective leucoanthocyanidin.

The biosynthesis of flavan-3-ols, the PAC precursors, is a long and complicate process involving three different pathways (shikimate, phenylpropanoid, and flavonoid pathways) and about 20 different enzyme-catalyzed reactions (Figure 5 and Figure 6) that occur on the cytosolic face of the endoplasmic reticulum (ER) of plant cells [21,22]. Therefore, the precursor units are transported into the vacuole where polymerization process probably takes place, leading to the formation of PACs [23,24].

The shikimic acid pathway consists of seven different metabolic steps that allow the biosynthesis of folates and aromatic amino acids, such as phenylalanine, tyrosine, and tryptophan [22,25]. The first reaction of this pathway is catalyzed by the 3-deoxy-D-arabinoheptulosonate 7-phosphate (DAHP) synthase (EC 2.5.1.54), which, starting from phosphoenolpyruvate (PEP) and erythrose-4-phosphate, leads to the formation of DAHP. DAHP is then converted into 3-dehydroquinate (DHQ) in a reaction catalyzed by the DHQ synthase (EC 4.2.3.4) that uses an NAD molecule as a cofactor. The subsequent two reactions involve the removal of a water molecule via the DHQ dehydratase (EC 4.2.1.10) using NADPH as a cofactor and forming 3-dehydroshikimate (DHS), and the reduction of the carbonyl group to the hydroxyl group by the activity of the shikimate dehydrogenase (EC 1.1.1.25) that allows the formation of shikimate. Therefore, shikimate is phosphorylated in position three by the shikimate kinase (EC 2.7.1.71), and condensed with 5-enolpyruvylshikimate-3-phosphate (EPSP) by the EPSP synthase (EC 2.5.1.19). The last reaction of the shikimate pathway, catalyzed by the chorismate synthase (EC 4.2.3.5), converts EPSP in chorismate that is the fundamental intermediate for the production, not only of all aromatic amino acids, but also of other non-amino acid aromatic compounds. However, in order to achieve the biosynthesis of PACs, it is necessary that chorismate is transformed into phenylalanine. Consequently, chorismate mutase (EC 5.4.99.5) catalyzes a Claisen rearrangement forming prephenate, which in turn is both decarboxylated in phenylpyruvate by the prephenate dehydratase (EC 4.2.1.51) and transaminated in phenylalanine (PHE) by the phenylpyruvate aminotransferase (EC 2.6.1.64) that transfers the amino group from a molecule of glutamic acid [25].

The four subsequent reactions are part of the phenylpropanoid pathway and allow the transformation of PHE into 4-hydroxychalcone, the key molecule at the beginning of the flavonoid pathway. This series of chemical reactions is made possible thanks to the activity of four cytosolic enzymes associated in a single multi-enzymatic complex anchored to the cellular RE through a N-terminal domain of one of these enzymes [26]. In particular, phenylalanine ammonia-lyase (PAL) (EC 4.3.1.24) cleaves the carbon–nitrogen bond of PHE using 4-methylideneimidazole-5-one (MIO) as a cofactor, and then converts it into trans-cinnamic acid. The previously formed cinnamic acid is then processed by the trans-cinnamate 4-monooxygenase (C4H) (EC 1.14.14.91), which is an enzyme belonging to the family of oxidoreductases, and able to add a hydroxyl group in para position of the ring. The last two reactions involve the combined action of 4-coumarate-CoA ligase (4CL) (EC 6.2.1.12) and chalcone synthase (CHS) (EC 2.3.1.74) that condensate and cyclize three malonyl-CoA molecules with 4-coumaroyl-CoA, leading to the formation of 4-hydroxychalcone (or naringenin chalcone) and, thus, starting the flavonoid pathway.

The flavonoid pathway is well known to be highly branched and complex. Indeed, through this pathway, the flavonoid scaffold can be largely modified, leading to the biosynthesis of almost all of the phenolic compounds thus far identified [27,28]. Below, the reactions involved in the synthesis of leucoanthocyanidins, the key PAC precursor compounds, will be described. The first step that characterizes this pathway is the isomerization of naringenin chalcone to naringenin, through the action of a ubiquitous enzyme named chalcone isomerase (CHI) (EC 5.5.1.6). Moreover, the isomerization of naringenin chalcone is a spontaneous reaction that could occur without the intervention of any enzyme. However, CHI stereospecifically directs and highly accelerates the cyclization of naringenin chalcone, thus facilitating and increasing the production yield of this intermediate fundamental for all subsequent biosyntheses [29]. Consequently, since the reaction catalyzed by CHI is highly stereoselective, the production of 2R-naringerin, which might occur from spontaneous isomerization, is drastically reduced [29]. Different enzymes can modify the naringenin, but only flavanone 3-dioxygenase (F3H) (EC 1.14.11.9), flavonoid 3’-monooxygenase (F3′H) (EC 1.14.14.82), and flavonoid 3’,5’-hydroxylase (F3′5′H) (EC 1.14.14.81) lead to the synthesis of flava-3-ol compounds. These three enzymes are oxidoreductases that selectively add one or two hydroxyl groups to naringenin. In particular, F3′H and F3′5′H add one or two hydroxyl groups to the B-ring of the flavanone scaffold leading to the formation of eriodictyol or tricetin, respectively. On the other hand, F3H adds a hydroxyl group to the C-ring of eriodictyol, tricetin, or naringenin leading to the biosynthesis of dihydroquercetin (DHQ), dihydromyricetin (DHM), or dihydrokaempferol (DHK), respectively. Moreover, since the reaction catalyzed by F3H is highly stereoselective, in this case, the formation of 3R-flavonols is limited [8,30]. If from a biosynthetic point of view F3H is fundamental for the formation of flavan-3-ols, F3’H and F3’5’H are two very important enzymes for the variability of PACs within plants. Indeed, the presence or absence of the gene sequences coding for these two enzymes strongly influence the hydroxylation pattern of B-rings of flavan-3-ols that will constitute the PACs as monomers [31,32,33].

The last step before the formation of leucoanthocyanidins involves the reduction of dihydroflavonols (DHQ, DHM, and DHK) by the action of the dihydroflavonol 4-reductase (DFR) (EC 1.1.1.219). This enzyme also belongs to the oxidoreductase family, but, unlike the previous ones, it simply reduces the ketone group in C_4_ of the C-ring to hydroxyl group. For this reason, leucoanthocyanidins are also known as flavan-3,4-diols.

At this point, leucocyanidin, leucopelargonidin, and leucodelphinidin can be converted into their respective anthocyanins by the anthocyanidin synthase (ANS) (EC 1.14.20.4) (Figure 6). This reaction allows the formation of the key compounds that may alternatively enter into biosynthetic pathway of anthocyanins, in which the anthocyanin scaffold may be further modified through different enzymatic modifications, including methylation, acetylation, and glycosylation [15,33]. However, anthocyanins may be converted into the respective colorless 2R,3R-flavan-3-ols by the double reduction operated by the anthocyanidin reductase (ANR) (EC 1.3.1.77). Moreover, since this enzyme is able to saturate the cationic C-ring of the anthocyanin scaffold, it strongly stabilizes the molecules from a chemical point of view. In another pathway branch, leucoanthocyanidins can alternatively be converted into 2R,3S-flavan-3-ols by the leucoanthocyanidin reductase (LAR) (EC 1.17.1.3) without going through the anthocyanidin intermediate (Figure 6). Moreover, this last reaction is very important as it explains the occurrence of PACs and anthocyanins in plants from a phylogenetic point of view. Indeed, plants lacking ANS and ANR are able to produce PACs, but not anthocyanins; plants lacking LAR and ANR are able to produce anthocyanins, but not PACs; meanwhile plants having all the previously reported enzymes are able to produce both PACs and anthocyanins. Moreover, in this latter case, PACs may be composed by both 2R,3S and 2R,3R flavan-3-ols [33].

### 3.2. Transport of Proanthocyanidins

As previously mentioned, once the precursor units are formed, they are transported into the vacuole where the polymerization process probably takes place, leading to the formation of PACs [19,34]. Several studies have been performed with the aim to identify and describe the mechanism related to the transport of PAC precursors from the RE cytosolic face to plant vacuole, but until now, a precise transport mechanism of individual flavan-3-ol monomers has not been well identified [19,35,36,37]. However, several hypotheses have been proposed. (i) Since the RE surface is actively involved in the synthesis of PAC precursors, it has been proposed that vesicles budded from rough RE (RER) may be involved [36,37,38,39]. In this case, the vesicles may transport the flavan-3-ols to the cis-face of the Golgi apparatus, where their contents could be released into lumen. Here, the molecules may be transported across the trans-face of the Golgi, chemically marked and consequently sent to the vacuole. This process, known as the trans-Golgi network, is typical of both animal and plant cells for the intracellular transport, not only of primary, but also of secondary metabolites [36,37]. (ii) A second potential transport mechanism of flavan-3-ols involves glutathione S transferase (GST) mediated transport (GST-mT). GSTs are enzymes known to be associated with detoxification and antioxidant processes in both animals and plants [40,41]. In this context, they are able to conjugate the glutathione (GSH) to electrophilic and lipophilic compounds increasing their hydrophilicity and, consequently, facilitating their elimination. Nevertheless, other functions for GSTs have been suggested, such as their contribution to sequestration and intracellular transport of secondary metabolites, including anthocyanins [36,37]. However, despite that direct contribution of GST in transport to the vacuole of many flavonoids has been proven, no experimental evidences of transport of flavan-3-ols have been reported. However, given the structural similarity of flavan-3-ols to anthocyanins, this transport mechanism has not only been proposed, but also the putative amino acids involved in the interaction GST-flavan-3-ols have been theoretically identified, albeit molecular docking analysis were not performed [36,37]. Recently, Ricardo Pérez-Díaz and colleagues (2016) combined molecular docking with gene expression analysis, giving, for the first time, experimental insights of the transport of PAC precursors mediated by GSTs in grapevine [34]. (iii) Finally, the last potential mechanism for the transport of flavan-3-ols from cytosol to vacuole may be related to ATP binding cassette (ABC), multidrug, and toxic compound extrusion (MATE), and mammalian bilitranslocase (BLT) transporters [24]. These proteins are membrane transporters and, although scientific evidences on their direct involvement for flavan-3-ol transport has not been reported, some experimental results demonstrate that they are actively implicated in the intracellular trafficking of many other active metabolites [24].

### 3.3. Polymerization of Proanthocyanidins

If the transport mechanism from plant cytosol to vacuole has only been theorized, the process by which the flavan-3-ols polymerize is even more ambiguous. Indeed, it is not yet clear whether a specific enzyme is involved in the regulation of the polymerization or if it may occur completely in a spontaneous way [19]. Recently, it was theorized that LAR could have a central role in the extension process of PACs. In particular, Liu and colleagues, studying the effects derived from the knockdown of *LAR* on the model legume *Medicago truncatula*, observed (i) a loss of low molecular weight PACs; (ii) a concomitant increase of PACs with high polymerization degree; (iii) a strong accumulation of 4β-(S-cysteinyl)-epicatechin. Consequently, the authors hypothesized that LAR may convert 4β-(S-cysteinyl)-epicatechin into epicatechin, the starter unit for PAC elongation [42]. Another crucial point for the polymerization of PACs concerns the possibility that the carbocation-form of flavan-3-ols can be synthesized in some way [43]. The presence of a carbocation species of flavan-3-ols would increase the reactivity of the reaction intermediates, and could explain the polymerization of PACs through non-enzymatic mechanisms. However, this is only another hypothesis and, in order to better understand this process, more biochemical and genetic evidences are needed.

## 4. Role in Plants

The main role of PACs in plants is represented by the first biochemical defense to external injuries (Table 1). Indeed, since plants are sessile organisms, they are subjected to a series of menaces derived not only from adverse environmental conditions, but also from animals, insects, fungi, bacteria, or other plants. Generally, these phenomena lead to the overproduction of reactive oxygen (ROS) and nitrogen (RNS) species, and then in oxidative stress [44]. ROS and RNS are very dangerous molecules for both animal and plant cells, as they are highly reactive and capable of compromising the normal function of a large class of biomolecules, including proteins, lipids, and nucleic acids [15,44]. In order to counteract the overproduction of ROS and RNS, during both biotic and abiotic stresses, the normal physiological functions of plants are alternated, and in particular, specific metabolic pathways are activated, resulting in the biosynthesis of both non-enzymatic antioxidants, such as ascorbic acid, flavonols, glutathione and various pigments, and/or enzymatic defenses [15]. Plant cells, unlike animal ones, are characterized by the presence of a large central vacuole where antioxidant flavonoids are accumulated, including PACs [45]. Moreover, as already described in the previous paragraphs, it is reasonable thinking that the elongation of PACs takes place inside this cellular organelle, despite that the polymerization mechanism is still unknown. The elongation of flavan-3-ol monomers into more complex molecules, such as PACs, is likely a strategy adopted by plants to increase the antioxidant properties of these molecules. This hypothesis is supported by experimental data through which the lower antioxidant capacity of monomers with respect to PACs have been demonstrated [45].

From a histological point of view, PACs are almost exclusively stored in the endothelial layer of the seed coats and in the epidermis and vascular bundles of plant leaves, thus constituting a protective barrier. Indeed, from these localizations, they can easily counteract both abiotic and biotic injuries [81]. The changes in PAC content under abiotic stress are more studied than those derived from biotic stresses (Table 1). However, while biotic stresses always result in an increasing of PACs, for abiotic stresses, the situation is more complicated. Indeed PAC biosynthesis and/or degradation seems to be dependent on both the type of stress and the plant species. Concerning biotic stresses, the astringent flavor determined by PACs is certainly one of the most common plant defenses against attacks by herbivores [82]. However, PACs have also been shown effective against fungi infection. Indeed, several experimental studies have reported that after the inoculation of different fungal strains, the plants increased the biosynthesis of PACs, causing a reduction, and in some cases the complete eradication, of the infection [71,72,73,74,75,76,77,78]. Similar phenomena were also observed during insect attack [79,80].

## 5. Analytical Methods for the Identification and Quantification of Proanthocyanidins

Despite the importance of PACs, an accurate and standardized method for their quantification is missed. Numerous analytical procedures, including colorimetric, gravimetric, chromatographic, and mass-spectrometric methodologies, are employed in order to detect, identify, or quantify PACs in plant samples. However, their extreme complexity and structural heterogeneity result in highly variable results. Below, the most employed methodologies will be discussed, describing experimental protocols and focusing on the main advantages and limitations.

### 5.1. Gravimetric Methods

Gravimetry is an old-fashioned method based on the selective separation of the compounds compound via precipitation or chromatographic separation. Despite gravimetric methods providing accurate data on the total content, they do not provide reliable qualitative information. Furthermore, although not expansive, gravimetric procedures are very long and complex. Consequently, their industrial applications are not appreciated [83,84,85].

Gravimetric methods consist of a number of purification steps by which the plant extract is fractionated using chromatographic columns and/or resins. Finally, the eluate is dried and the solid residue is weighed. For PAC quantification, the plant raw material is normally extracted until exhaustiveness using a variable extraction ratio, ranging from 1:10 (*w/v*) to 1:100 (*w/v*). The obtained extract is then fractionated via reverse chromatographic resins and finally weighted.

**Figure 7:**
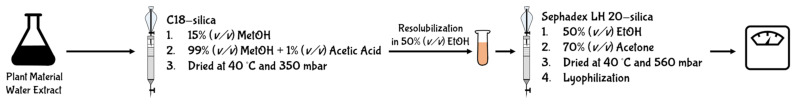
Schematic representation of the gravimetric method for the quantification of PACs.

Experimentally, in order to remove sugars and organic acids that may be present in the aqueous extract, the sample containing PACs is loaded into a reverse phase C18-silica column. At the beginning, water is flushed into the column alone, and then with 15% (*v/v*) methanol. After the hydroalcoholic solution is completely eluted, 99% (*v/v*) methanol acidified with 1% (*v/v*) acetic acid is added to detach polyphenol compounds from the column. The fraction containing polyphenol compounds is then dried at 40 °C and 350 mbar using a rotary evaporator. The obtained dried extract is resuspended in 50% ethanol (*v/v*) and again loaded into a chromatographic column packed with an adsorption resin (Sephadex LH-20). In order to remove polyphenolic glycosides without eluting PACs, 50% (*v/v*) ethanol is added to the column. PACs are therefore detached from the column by adding 70% (*v/v*) acetone. The organic solvent of this last fraction is completely removed via a rotary evaporator set up at 40 °C and 560 mbar, meanwhile the residual water content is freeze-dried. The obtained dried extract is weighed and compared to the starting sample weight (Figure 7) [85].

### 5.2. Colorimetric Methods

Unlike gravimetric methodologies, colorimetric assays are not only easy to perform, they are low-cost procedures. These methodologies are mainly divided into two groups: (i) spectrophotometric methods based on PAC hydrolysis into anthocyanins; and (ii) complexation reactions with chemical reagents. In the first case, the measurement of the absorbance is performed at the typical wavelength of anthocyanidin compounds (λ = 510–520 nm), whereas the complexation reactions typically produce a bathochromic shift into wavelengths in which few, or none, interferences are recorded. Consequently, the methodologies based on PAC hydrolysis can be highly influenced by the basal content of anthocyanin compounds present in the raw material, resulting in unreliable measurements. On the contrary, the methodologies based on the bathochromic shift allow to drastically reduce this interference.

#### 5.2.1. Acid Butanol Assay

**Figure 8:**
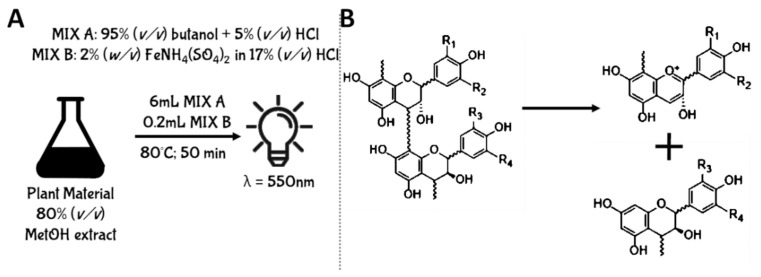
Schematic representation of Acid Butanol Assay for the quantification of PACs. Panel (**A**) displays the experimental protocol; Panel (**B**) displays the chemical reaction that allows the formation of the carbocation that spontaneously and quickly arrange in the respective anthocyanin.

One of the main characteristics of PACs is related to their peculiar ability to depolymerize in both acid and strong oxidizing environments leading to the formation of the respective anthocyanin compounds [86]. Consequently, the uncolored mixture containing PACs assumes a very intense red coloration. This unusual property of PACs was then exploited for the development of analytical methods aimed at their quantification. In this context, the Acidic Butanol Assay (also known as Porter’s method or Bate-Smith Assay) is a spectrophotometric assay experimentally designed to quantify PACs using the absorbance produced by anthocyanins derived from their depolymerization process [86]. This methodology consists of the preparation of a reaction mixture, composed of 95% (*v/v*) butanol acidified with 5% (*v/v*) HCl (Reagent A), and of a catalytic mixture containing 2% (*w/v*) FeNH_4_(SO_4_)_2_ dissolved in water acidified with 17% (*v/v*) HCl (Reagent B). Concerning the experimental protocol, the plant raw material is normally extracted in 80% (*v/v*) methanol using a ratio ranging from 1:5 (*w/v*) to 1:20 (*w/v*). Then, to 1 mL of plant extract are added 6 mL of Reagent A and 200 μL of Reagent B. Therefore, the mixture is centrifuged (8000× *g*, at room temperature) and incubated at 80 °C for 50 min. During the incubation time, the interflavan bonds are cleaved, forming highly unstable intermediates named carbocations. Since these compounds are very unstable, they spontaneously and quickly arrange in the respective anthocyanins [86]. Once the anthocyanins are formed, the mixture is cooled for 25 min at room temperature, and the absorbance is read at 550 nm (Figure 8).

However, several limitations were reported for this assay: (i) since the depolymerization of PACs generate different typologies of anthocyanins with specific maximum wavelength of absorbance, the qualitative composition of PACs strongly affect the spectrophotometric assay [87]; (ii) increased concentrations of transition metal ions catalyzing the color formation decrease the color development and the depolymerization process [88]; (iii) the formation of anthocyanin compounds interferes in PAC quantification for plant extracts that simultaneously contain both PACs and other red colored pigments, like anthocyanins or betalains. Consequently, the acid butanol assay should be used with caution if quantitative results must be provided [89,90]. On the other hand, despite that quantitative results cannot be accurately provided using this method, it is useful to provide information regarding the presence or absence of PACs in plant extracts [89].

#### 5.2.2. Pharmacopoeia Method

**Figure 9:**
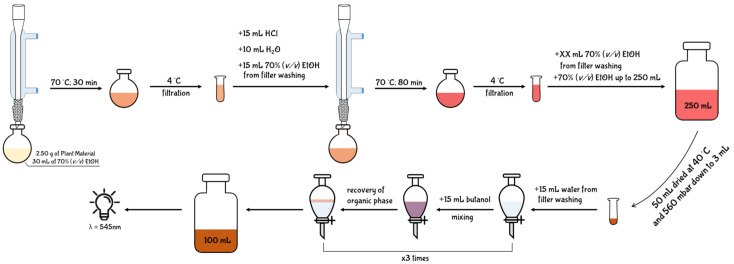
Schematic representation of the Pharmacopoeia Method employed for the quantification of PACs from Crataegus fruits.

In the second volume of the European Pharmacopoeia, an analytical assay for the quantification of PACs from extracts of Crataegus fruits is described [91]. Since the aim of the European Pharmacopoeia chapter is to provide a quality code, no indication regarding therapeutic activity, toxicity, or dosage is reported for PACs. Despite the reliability of Pharmacopoeia for the quality control of pharmaceutical products, some of the assays described for the quantification of phytochemicals are quite dated and approximate. In particular, the assay reported for the quantification of PACs is a long, complex, and expensive method that leads to the collection of unreliable results [92] (Figure 9).

The experimental protocol described in the Pharmacopoeia reports that 2.50 g of plant raw material is weighed and extracted with 30 mL of 70% (*v/v*) ethanol. Consequently, the mixture is heated to 70 °C under reflux using a round-bottom flask combined with a condenser tube. After 30 min, the extract is cooled on ice and filtered on filter paper. In order to recover any residues from the filter, 10 mL of 70% (*v/v*) ethanol are employed for washing. The washing solvent is then added to the extract, and the mixture is acidified with 15 mL of HCl and diluted with 10 mL of water. The new acidic mixture is again heated to 70 °C for 80 min under reflux using a clean round-bottom flask combined with the same condenser tube. After the incubation time, the mixture is again cooled on ice, and filtered with a clean paper filter. Moreover, in this case, the filter is washed with 70% (*v/v*) ethanol until it is completely whitened. The filtrate and the washing solvent are again combined and the mixture is diluted with 70% (*v/v*) ethanol up to a final volume of 250 mL. Only a 50 mL aliquot of the diluted mixture is concentrated down to 3 mL under reduced pressure using an evaporating rotator (40 °C, 350 mbar). Therefore, the concentrated mixture is transferred into a separatory funnel, and the round-bottom flask is sequentially washed with 10 mL and 5 mL of water. The resulting 15 mL of washing solvent is then combined in the separatory funnel with the mixture previously concentrated. Finally, in order to perform a liquid/liquid separation, 15 mL of butanol are loaded into the separatory funnel and then vigorously shaken for few seconds. After a rapid decantation, the butanol phase enriched in anthocyanins derived from PAC hydrolysis separates from the aqueous one. The butanol phase is then collected and transferred in a clean glass-cylinder. The separation process with butanol is repeated twice, and the individual organic phases are then combined in the same glass-cylinder. The obtained 45 mL are then diluted up to 100 mL with pure butanol, and the absorbance of 1mL of the mixture is read at 545 nm. The content of PACs is finally expressed as percentage using Equation (1):

%PACs = (A × 500)/(75 × m),(1)

where: ‘A’ is the absorbance recorded at 545 nm for one mL of the mixture, and ‘m’ is the weight of the starting plant material used for extraction and expressed as grams.

#### 5.2.3. Vanillin Assay

**Figure 10:**
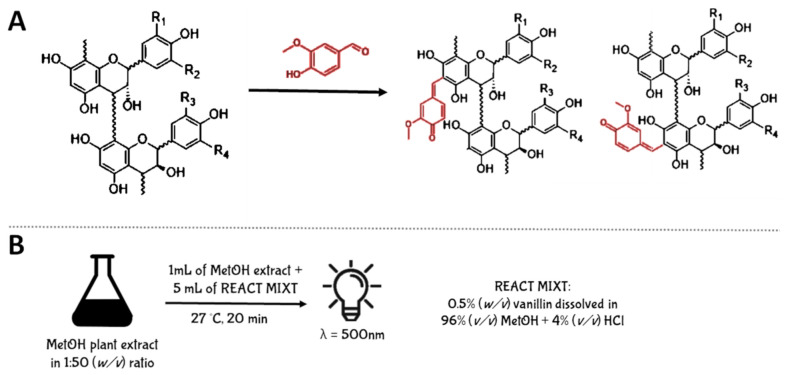
Schematic representation of Vanillin Assay for the quantification of PACs. Panel (**A**) displays the chemical reaction that allows the formation of red colored adducts, spectrophotometrically measured at 500 nm. Panel (**B**) displays the experimental protocol.

The first method employed for PAC quantification and not based on their depolymerization is the Vanillin Assay. This assay involves the condensation of vanillin, an aromatic aldehyde, with the hydroxyl group present on C_6_ of the A-ring of the flavan-3-olic scaffold. The reaction yields the formation of a red colored adduct that is spectrophotometrically measured at 500 nm [93] (Figure 10A).

Experimentally, the plant raw material is extracted with methanol using 1:50 (*w/v*) ratio. After centrifugation and filtration, 5 mL of reaction mixture, composed by 0.5 (*w/v*) vanillin solubilized in 96% (*v/v*) methanol acidified with 4% (*v/v*) HCl, are added to 1 mL of plant extract. Therefore, it is incubated at 27 °C for 20 min and the absorbance is read at 500 nm against a blank not containing the plant extract. The quantification is performed using catechin for the construction of a calibration curve, and the results are expressed as mg of catechin equivalent (EC) per 100 g of plant material (Figure 10B).

Despite Vanillin Assay being a fast and inexpensive method, it has several limitations. In particular, (i) it was demonstrated that the condensation reaction is not specific for PACs, since properly substituted compounds including dihydrochalcones, anthocyanins, flavan-3-ols and ascorbic acid, can also react with vanillin causing an overestimation of the PAC content [94]; (ii) the formation of a red colored adduct may interfere with PAC quantification in plant extracts that simultaneously contain both PACs and other red colored pigments, like anthocyanins or betalains [94]; (iii) the acidity of the extraction and reaction solvent strongly influences the kinetics of the condensation reaction, resulting in the production of greater color intensities [94,95]; (iv) the presence of water in the plant sample also negatively influences this reaction; (v) excessive concentrations of vanillin in the mixture yields to a self-condensation process causing an error in the PAC quantification [94,95]; (vi) small changes in reaction temperature lead to important variations in absorbance [95].

#### 5.2.4. Brunswick Laboratories 4-dimethylaminocinnamaldehyde (BL-DMAC) Assay

BL-DMAC is a colorimetric assay known to be the most accurate method for PAC estimation. Originally, the assay was studied with the aim to detect and quantify PACs from cranberry samples and correlate their content with potential antimicrobial activity against uropathogenic *Escherichia coli* [83]. However, after several experimental studies, the high reproducibility and analytical precision of BL-DMAC was demonstrated, also using different typologies of plant raw materials [96,97,98,99,100,101,102] and their derived products [47,64,103,104,105]. Since the PAC determination occurs at 640 nm, this assay is less affected by the presence of other phytochemicals, including anthocyanins [83]. However, the chemical reaction that allows the bathochromic shift of PACs from 260 to 640 nm is not well known. It is hypothesized that in an acidic environment the aldehyde group of the DMAC molecule is protonated, leading to the formation of a highly reactive carbocation. This carbocation specifically reacts with molecules (1) having hydroxyl groups in meta-position of the A-ring of the flavonol scaffold; (2) having a single bond C_2_–C_3_; and (3) not having a carbonyl at C_4_ [96]. Consequently, in addition to PACs, only flavan-3-ols (such as catechins and epicatechins) and some anthocyanins (such as cyanidins and delphinidins) can react with DMAC reagent, causing a potential interference, which was proven to be really weak [96].

**Figure 11:**
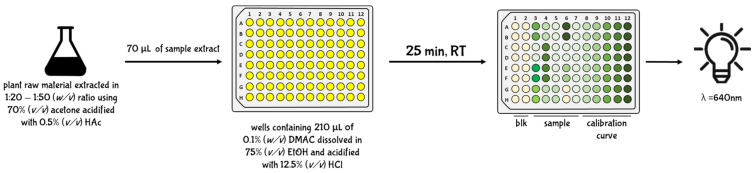
Schematic representation of BL-DMAC assay for the detection and quantification of PACs.

Experimentally, the plant raw material should be extracted with 75% (*v/v*) acetone acidified with 0.5% (*v/v*) acetic acid and using 1:20–1:100 (*w/v*) ratio. The mixture is then vortexed for 30 s, sonicated at room temperature for 30 min, and placed on an orbital shaker for 60 min. After centrifugation (2000× *g* at room temperature for 10 min), 70 μL of a proper dilution of the extract is added to 210 μL of DMAC solution containing 0.1% (*w/v*) DMAC dissolved in 75% ethanol (*v/v*) acidified with 12.5% (*v/v*) hydrochloric acid. After 25 min of incubation, the absorbance is read at 640 nm and against a blank containing 70 μL of extraction solvent and 210 μL DMAC solution. PAC content is expressed and mg A-type PAC equivalents per 100 g of fresh weight using a calibration curve of pure PAC standard ranged between 20 and 100 ppm (Figure 11).

### 5.3. Mass Spectrometry (MS) Methods

Unlike other polyphenolic compounds, the quantification of the punctual PACs through mass-spectrometry (MS) methodologies is still under investigation and currently represents a hard challenge. Indeed, the analytical process is strongly affected from multiple factors, including: (i) the great qualitative heterogeneity of the monomers that constitute PACs; (ii) the variable number of monomeric subunits that can be present in PAC structures (from 2 to 60 units); (iii) the lack of commercially available standards fundamental for their analytical quantification. For these reasons, the UV/Vis methodologies previously described and aimed to the quantification of the total PAC amount are still widely used despite providing data considerably affected by the different experimental conditions used. On the other hand, MS-based methods could give a more precise and standardized information of PAC profile. However, both MS methods coupled with liquid chromatography (LC) or with matrix-assisted laser desorption ionization (MALDI) have severe limitations.

#### 5.3.1. Chromatographic System

LC–MS methods for PAC quantification consist in the separation of these molecules using chromatographic columns. However, plant extracts containing PACs are complex mixtures of other phytochemicals and PACs, having several and different polymerization degrees [106]. It was reported that PACs with a polymerization degree ranging between 2 and 10 can be efficiently separated via normal phase chromatography. However, PACs with a polymerization degree higher than 10 co-elute all together at the end of chromatographic run [107]. Moreover, an additional problem with the use of normal phase chromatography is the interference caused by the co-elution of other phytochemicals during the chromatographic run. For this reason, chromatographic methods employing the normal phase are currently rare and replaced by reverse phase chromatography [107,108,109]. However, even if reverse phase columns can easily fractionate monomers, dimers, trimers, and tetramers of PACs and their relative isomers, the order of elution is not in accordance with their molecular size. It has also been reported that the analysis of PACs with polymerization degree higher than tetramers is strongly affected by the co-elution of PAC oligomeric isomers. Indeed, reversed phase columns are able to separate oligomers of equivalent molecular mass into their isomers, but proanthocyanidins bigger than tetramers have a large number of isomers which elute together causing an overlap of the retention time. Consequently, isomers of the same oligomers are recorded in the chromatogram in a single and large unresolved peak that cannot be neither identified and/or quantified [110].

Additionally, UV/Vis detectors are avoided due to the non-specific maximum wavelength of PAC absorbance (280 nm). On the other hand, fluorescence detectors, although offering increased sensitivity and selectivity for some PAC typologies, show similar problematics. Moreover, fluorescence quantification is also affected by the qualitative composition of PACs that strongly modifies the emission and excitation maximum wavelengths [108]. Consequently, mass spectrometry (MS) detectors seem to be the only ones able to provide a realistic identification and quantification of PACs, although an additional limitation is related to the ionization methodologies. The development of electrospray ionization (ESI) had an enormous impact on the analysis of plant bioactive compounds, including PACs, achieving the simultaneous volatilization and ionization also for non-volatile molecules. However, ESI is not well suited for the analysis of highly variable molecules like PACs, because it generates several charged ions that make impossible spectra interpretation. Finally, the most common MS detectors coupled with LC–ESI instrumentations have a very limited range of molecular weight acquisition. The above mentioned problems explain why in literature no scientific articles reporting the quantification of PACs having polymerization degree higher than 10 are available.

#### 5.3.2. Matrix-Assisted Laser Desorption/Ionization (MALDI) System

Analysis of PACs using MS-based methods can alternatively be performed without solving the chromatographic separation problems. In this case, MALDI can be used as ionizing source and chromatographic co-elution problems are avoided [111]. Moreover, MALDI has a greater tolerance for impurities with respect to ESI. This system is able to detect mainly single-charged molecular ions, and is designed to interface with high resolution detectors, such as the time-of-flight (TOF) detector [111,112]. Indeed, unlike LC–MS instrumentations, the analysis performed via MALDI-TOF not only have unlimited mass range, but also higher sensitivity. Consequently, qualitative analyses on plant samples may include PACs with very high polymerization degrees [113,114]. However, quantification by MALDI-TOF is still a great challenge because pure analytical standards are not commercially available. Indeed, similar to other MS detectors, also during TOF analysis the equimolar loading of different compounds results in peaks of different intensity, which cannot be quantified in a semi-quantitative fashion. Moreover, MALDI-TOF spectra generated from the loading of the same sample have a strong variability in ion current, noise level, baseline, and peak intensities. These variations are equally present after consecutive laser shots in the same position or across different locations of the target surface. Finally, competitive ionization/ion suppression is an additional factor that hinders this kind of analysis, especially in complex samples such as plant material [114].

Therefore, although MALDI-TOF allows to obtain a truly representative profile of PACs theoretically including all existing polymerization grades, quantifications through this MS technique is not recommended [114].

## 6. Distribution in Edible Sources

PACs are bioactive compounds variously distributed within the plant kingdom [115]. Most of red fruits are well known to contain high contents of PACs; however, their presence has also been detected also in non-red colored roots, leaves and fruits [115]. The simultaneous presence of anthocyanins and PACs in red fruits can be explained by the ability of the plants to regulate the transcription of genes encoding for ANS and ANR, which respectively catalyze the transformation of leucoanthocyanidins into the respective anthocyanins, and their consequent reduction into 2R, 3R-flavan-3-ols. On the contrary, plants having PACs only, lack both ANS and ANR, but express LAR that directly yields to the production of 2R, 3S-flavan-3-ols from leucoanthocyanidins (Figure 6).

In order to investigate the distribution of PACs in the plant kingdom, a database consisting of published articles in which the phytochemical composition of different plant raw materials containing PACs was built. This selection provided 3868 entries, which were individually analyzed to select papers that provided PAC estimation through BL-DMAC assay (*n* = 41). Then, information regarding the species binomial name, plant family, common name, and plant part used for the extraction was extrapolated along with the PAC content. Data reported using different measurement units were homogeneous, fixing for the water content when necessary. Consequently, all data were expressed as mg PAC equivalents per 100 g of fresh weight.

**Figure 12:**
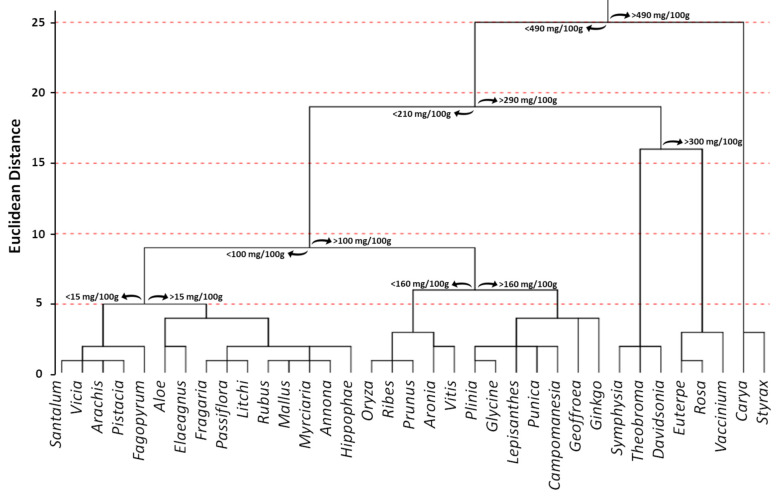
Cluster distribution of proanthocyanidins based on the total proanthocyanidin content within plant families and according to previously published data [96,97,99,100,101,102,104,105,116,117,118,119,120,121,122,123,124,125,126,127,128,129,130,131,132,133,134,135,136,137,138,139,140,141,142,143,144,145,146,147,148]. Euclidean distances were calculated with the average linkage method. Statistical analysis and graphical representation were made using SPSS v. 24 software.

The total number of selected species was 55, and the estimated average PAC content was 171.80 mg PACs per 100 g of fresh weight. The 55 species belonged to 35 different families (Figure 12). Among them, the most representative family was *Vaccinium*, which included 10 different species. Moreover, the *Vaccinium* family also displayed one of the highest PAC values with respect to the other families. In particular, it recorded a mean value equal to 290.97 mg PAC equivalents per 100 g of fresh weight, which was lower only to Styrax (497 mg PAC equivalents per 100 g of fresh weight) and Carya (508 mg PAC equivalents per 100 g of fresh weight) families. On the other hand, Santalum (10.5 mg PAC equivalents per 100 g of fresh weight), Vicia (10.2 mg PAC equivalents per 100 g of fresh weight), and Fagopyrum (2.5 mg PAC equivalents per 100 g of fresh weight) displayed the lowest ones (Figure 12).

**Figure 13:**
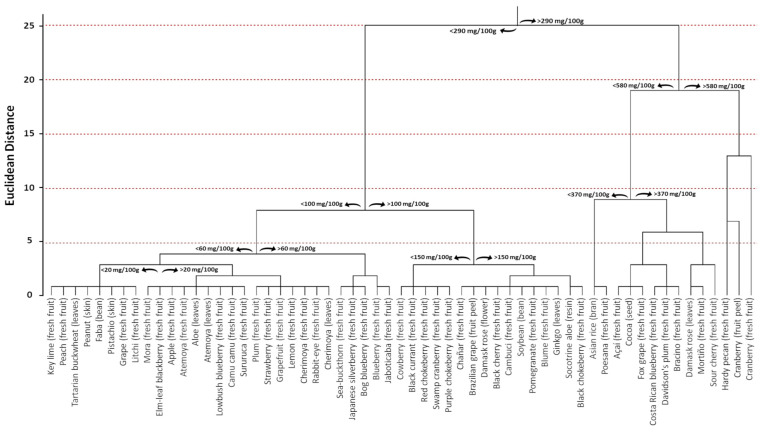
Cluster distribution of proanthocyanidins in plant kingdom based on the total proanthocyanidin content according to previously published data [87,88,90,91,92,93,95,96,107,108,109,110,111,112,113,114,115,116,117,118,119,120,121,122,123,124,125,126,127,128,129,130,131,132,133,134,135,136,137,138,139]. Euclidean distances were calculated with the average linkage method. Statistical analysis and graphical representation were made using SPSS v. 24 software. 7. PAC Availability.

Focusing on the plant raw material, the database contained the PAC estimation from four beans/seeds (*Glycine max*, *Vicia faba*, *Theobroma cacao*, and *Oryza sativa*), one flower (*Rosa damascena*), 43 fresh fruits (*Annona atemoya, Annona cherimola, Aronia arbutifolia, Aronia melanocarpa, Aronia prunifolia, Campomanesia phaea, Carya illinoinensis, Citrus aurantifolia, Citrus limon, Citrus paradisi, Davidsonia pruriens, Elaeagnus umbellata, Euterpe oleracea, Fragaria ananassa, Geoffroea decorticans, Hippophae rhamnoides, Lepisanthes alata, Litchi chinensis, Malus domestica, Myrciaria dubia, Passiflora setacea, Plinia cauliflora, Prunus cerasus, Prunus domestica, Prunus serotina, Punica granatum, Ribes nigrum, Rubus adenotrichus, Rubus ulmifolius, Santalum acuminatum, Styrax ramirezii, Symphysia poasana, Vaccinium angustifolium, Vaccinium ashei, Vaccinium consanguineum, Vaccinium cyanococcus, Vaccinium floribundum, Vaccinium macrocarpon, Vaccinium oxycoccos, Vaccinium uliginosum, Vaccinium vitis idaea, Vitis labrusca,* and *Vitis vinifera*), four fruit peels/skins (*Arachis hypogaea, Pistacia vera*, *Plinia cauliflora*, and *Vaccinium macrocarpon*), six leaves (*Aloe vera, Annona atemoya, Annona cherimola, Fagopyrum tataricum, Ginkgo biloba*, and *Rosa damascena*), and one resin (*Aloe perryi*) (Figure 13). The large number of samples related to fresh fruits is certainly linked to the importance that PACs have shown in producing beneficial effects on human health through the consumption of foods rich in these bioactive compounds. Indeed, it is well known that plants tend to synthesize and store bioactive compounds, including PACs, not only in fruits, but also in leaves and flowers [16]. However, since most flowers and leaves are not edible, the estimation of PAC content has been almost exclusively limited to fruits. Consequently, the limited availability of data related to the content of PACs in other plant parts is the major limitation of the analysis presented in this section (Figure 12 and Figure 13).

To assess potential positive effects of dietary phytochemicals on human health, the evaluation of their bioavailability is essential knowledge. Indeed, unless local effects at the bowel level are considered, a dietary compound must be available in the blood to exert biological effects. Therefore, bioactivity of phytochemicals in vivo depend on their release from the food matrix, their stability under digestion conditions, their intestinal absorption, their metabolism, and excretion [149]. On the other hand, potential biological activity of phytochemical metabolites must also be considered [150].

Physiochemical parameters largely influence phytochemical bioavailability [151]. In particular, molecular weight was shown to be a determining factor for intestinal absorption [152]. Moreover, some small polyphenols, such as phenolic acids, are easily absorbed through the gut barrier via paracellular or transcellular transport, eventually involving protein carriers [153,154]. On the contrary, for large molecular weight compounds, such as proanthocyanidins and ellagitannins, intestinal absorption is quite limited [149]. The uptake of proanthocyanidins at the proximal intestinal is rather poor, while at the level of the colon, almost exclusively proanthocyanidin dimers and trimers are effectively absorbed. Indeed, it was demonstrated by in vitro models of trans-epithelial transport and intestinal digestion that permeation coefficient across epithelial monolayer is a function of polymerization degree [155,156]. Accordingly, Ou et al. evaluating the transport of A-type PAC dimer, trimer and tetramer through in vitro Caco2 monolayer systems, recorded a transport ratio of 0.6%, 0.4%, and 0.2%, respectively [157]. Therefore, the bioavailability of PACs depends on the presence in the food matrix of small bioavailable oligomers or on the degradation of large proanthocyanidins to dimers and trimers during gastrointestinal digestion. Moreover, the low bioavailability of these large polyphenols is also due to their ability to interact with other components in the digestive tract, including food matrix components, intestinal mucosa constituents, and digestive enzymes [158]. The better known property of tannins is their capacity to form complexes with proteins [159]. This property may have nutritional implications. Indeed, PACs affect digestive processes by binding digestive enzymes and dietary proteins. For instance, PACs have been shown to interact with α-amylase producing complexes undetectable in plasma samples [160,161]. Additionally, it has also been demonstrated that high molecular weight PACs can interact with cell membranes [162,163]. In particular, large proanthocyanidins have been shown to produce a significant increase in the trans-epithelial electrical resistance (TEER) of polarized intestinal epithelium monolayers, which is indicative of their interactions with plasmatic membrane [164,165].

Regarding PAC stability in the stomach after the intake of proanthocyanidin rich-foods scientific data are very conflicting. Some in vitro studies showed PAC degradation under the acid conditions of the gastric environment. In particular, it was showed that large polymers produce a precipitate, while oligomers, until hexamers, are hydrolyzed to catechin and PAC dimers, which are able to cross intestine mucosa [150,166]. In contrast, Rios et al. demonstrated that in human subjects proanthocyanidins, regardless of the molecular weight, are remarkably stable in the stomach environment reaching high concentration in intact form in the small intestine [167]. Accordingly, Serra et al., using a combination of in vitro and in vivo murine models, showed a significant PACs stability at gastric digestive conditions [168]. Conversely, in a randomized cross-over study in humans, the demonstrated absence of proanthocyanidin monomers and dimers in plasma samples after intake of cocoa beans, which are high in large proanthocyanidins, suggests the lack of proanthocyanidin depolymerization during both gastric and intestinal digestion [169]. In contrast, evaluating bioavailability of proanthocyanidins from *Choerospondias axillaris* peels in an in vitro model of gastrointestinal digestion, Li et al. showed that the total polyphenol content and the mean degree of polymerization, unchanged during gastric digestion, suffered a strong reduction during intestinal digestion. The authors suggested that pancreatic enzymes, rather than the pH value of the intestinal milieu, could be involved in proanthocyanidin degradation [170]. However, other studies suggested a significant influence of pH in the intestinal environment on proanthocyanidin digestion and absorption. Indeed, it was assessed in an in vitro model of gastrointestinal digestion that large proanthocyanidins, producing precipitate under gastric conditions, were solubilized at the basic environment of the gut becoming available to subsequent modifications and absorption [156].

The susceptibility of PACs during intestinal digestion also depends on the intestinal microbiome. In particular, in a murine model, it was shown that although epicatechin can be produced by PAC degradation by colonic microflora, it is subsequently rapidly metabolized to low molecular phenolic acids that are the main metabolites of PACs in urine [152,171]. Moreover, Tao et al. proposed that proanthocyanidin dimers and trimers are the main substrates of the gut microbiota, while large polymers are less fermented substrates [156].

Based on what is known about digestive stability and absorption of proanthocyanidins, it is therefore likely that the systemic effects of these phytochemicals have to be attributed to the small oligomers (dimers and trimers). On the contrary, high molecular weight proanthocyanidins, rather stable under gastrointestinal digestion conditions and little susceptible to fermentation by the microflora of the colon, as a consequence of the poor ability to cross the intestinal barrier, reach high concentrations in the colon in the native form, potentially useful to carry out local action at the gut level. On the other hand, these effects could be useful in explaining the protective effects of PACs on the physiology of the intestinal tract, including the inverse correlation between the intake of foods rich in proanthocyanidins and the risk of colorectal cancer [172].

## 7. PAC Bioactivity

Through their well-established antioxidant properties discussed above, PACs may exert crucial roles in several pathological conditions, including cancer, inflammatory, cardiovascular and neurodegenerative diseases and metabolic disorders. Indeed, all these pathological processes may take off severe conditions of oxidative stress (OS) and PACs may tone down OS both by acting as free radicals’ scavengers and by affecting signaling pathways associated with cellular OS homeostasis. Among them, the best characterized pathways that have been shown to be influenced by PACs in several human, animal, and culture studies are those involving nuclear factor erythroid 2-related factor 2 (Nrf2), mitogen-activated protein kinase (MAPK), nuclear factor-kB (NF-κB), and phosphoinositide 3-kinase/protein kinase B (PI3K/Akt).

In recent years, the molecular events and signaling pathways involved in the antioxidant mechanism of specific PACs have been extensively investigated and partly clarified, increasingly emphasizing the potential of these molecules in the clinical setting for the prevention and treatment of various OS-associated diseases. Moreover, PACs have been shown to be safe and to have not apparent side effects, thus making them suitable, promising and powerful candidates in clinical medicine.

Many studies on PAC’s benefits on human health have been published and, recently, well summarized, in particular regarding their anti-cancer, cardiovascular, and neurological protective properties [30,43]. Moreover, emerging evidence from clinical studies indicates that higher PAC intake is associated with reduced risk of several metabolic disorders, including metabolic syndrome (MetS), non-alcoholic fatty liver disease (NAFLD), and non-alcoholic steatohepatitis (NASH), type 2 diabetes mellitus (T2DM), and the complications associated with it, such as nephropathy and neuropathy [173,174,175]. In particular, PACs are emerging to play a key role in modulating glucose- and lipid-lowering effects. In this review, we will mainly focus on the role of PACs in metabolic disorders, delving into the mechanisms through which this class of compounds may affect both glucose and lipid metabolism also thanks to their capability to interact with food-derived proteins, digestive enzymes, and cell membrane proteins along the entire gastrointestinal tract [176].

### 7.1. Glucose-Lowering Effect

**Figure 14:**
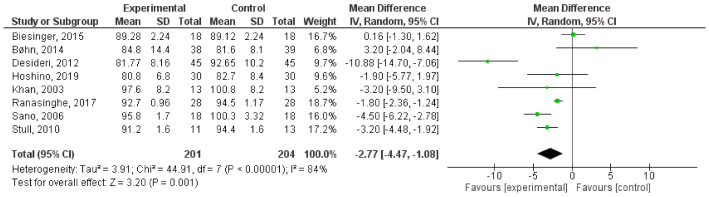
Forest plot representation of the effects derived from the supplementation of PACs on hematic sugar levels. Data were extrapolated from [177,178,179,180,181,182,183,184], and plotted according to the mean difference. Each horizontal line of the plot represents an individual study, reporting the punctual result plotted as green box. The weight of each study is represented by the size of the green box. The horizontal line indicates the lower and upper limit of the 95% Confidence Interval (CI) of the effect observed for each study. The vertical line represents the no-effect. For each study, if the horizontal line crosses the vertical one, a statistically significant difference between Experimental and Control group is not observed. The black diamond at the bottom of the forest plot represents the average effect size combining together the results of all the selected studies. The horizontal points of the diamond are the limits of the 95% CI of the average value. The figure was generated by Review Manager Software, version 5.4.1.

In order to understand if PAC supplementation could affect blood glucose levels, we performed a meta-analysis on data collected from articles published in the last 10 years and that satisfied the pre-established inclusion criteria. Briefly, the previously published articles (*n* = 327) were obtained by a literature search on PubMed, Scopus, Google Scholar, and ISI Web of Science research tool using the following keywords: (“proanthocyanidin(s)” OR “procyanidin(s)” OR “PAC(s)” AND “blood glucose” OR “glucose” OR “glycaemia”). Then, a manual screening of the articles was performed by reading the title, abstract or full text. Original articles were exclusively included if they met the following inclusion criteria: (i) the language should be English; (ii) articles should be published in peer-review journals; and (iii) after the reviewing by experts; (iv) the study design should be a randomized controlled clinical trials on human; (v) the intervention should be the supplementation of formulation containing PACs only, not in combination with other substances; (vi) only studies where the number of participant has been clearly reported should be included; (vii) the parameter measured should be the blood glucose level; (viii) when outcomes were presented at different times in the study, only the longest follow-up duration was selected. Accordingly, of the 327 published full text articles that were identified during the bibliographic research, 319 were excluded. Data from the selected articles (n = 8) were employed for the meta-analysis [177,178,179,180,181,182,183,184]. Since data were accumulated from a series of studies that had been independently performed, all of the selected studies were not functionally equivalent. Consequently, the originated forest plot (Figure 14) was performed using random effect, according to the heterogeneity calculated between the studies. Statistical heterogeneity among studies was checked with the Cochrane Q test (significance level of *p* < 0.05) and the I^2^ statistic.

The combined results of the selected articles from the random-effect model suggested a significant effect of PAC supplementation on blood glucose levels (WMD: −2.77 mg/dL; 95% CI: −4.47, −1.08; I^2^ = 84%; *p* = 0.001). Furthermore, sensitivity analyses were performed to evaluate the influence of each study on the overall effect size. Finally, potential publication bias was checked by visual inspection of the respective funnel plot. As Supplementary Figure S1A displays, no publication bias was identified among the selected studies.

**Figure 15:**
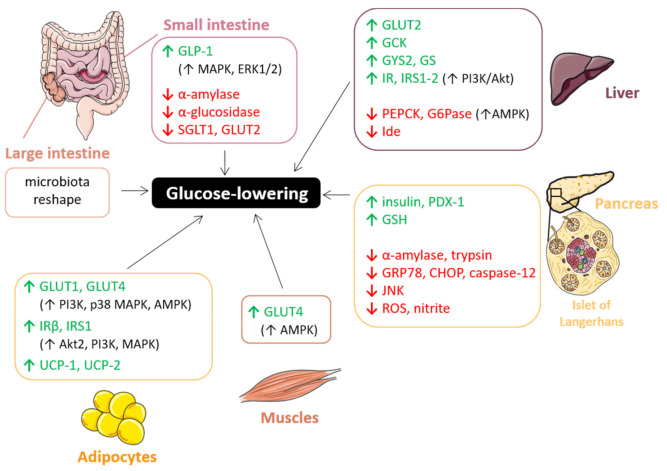
Schematic representation of the molecular mechanisms through which PACs affect glucose metabolism protecting against hyperglycemia. **↑** increase; **↓** decrease. The figure was created using Servier Medical Art by Servier (smart.servier.com, accessed on 12 March 2021), licensed under a Creative Commons Attribution 3.0 Unported License).

In the next subsections, we will deepen the potential beneficial effects of PACs on hyperglycemia sustained by several in vivo studies (Table 2). In particular, we will investigate the major molecular mechanisms by which PACs can interfere with metabolic glucose signaling at different levels and in different target organs, including the small and large intestine, liver, pancreas, skeletal muscle, and adipose tissues (Figure 15).

#### 7.1.1. Gut: Carbohydrate Digestion and Glucose Absorption

Complex carbohydrates, once reached the small intestine, are mainly digested by α-amylase and α-glucosidase, two key carbolytic enzymes involved in post-prandial glycemic response, which convert them into monomers. The latter are then incorporated by enterocytes through specific transporters localized at the apical side of their brush border membrane. Among them, sodium-dependent glucose transporter (SGLT1) and glucose transporter GLUT2are inhibited by PACs [215], thus preventing glucose absorption. Glucose tolerance was also found to be favored by PACs thanks to their capability to promote, both in vitro and ex vivo, the secretion of glucagon-like-peptide-1 (GLP-1), one of the most important satiety-related enterohormones: grape seed proanthocyanidins extracts (GSPE) stimulate GLP-1 secretion in the ileum, whereas unabsorbed or metabolized forms do the same in the colon probably through MAPK and ERK1/2 pathways [216,217]. The suppression of GLP-1 secretion seems to be dependent from PAC concentration and its subsequent effect on cellular membrane potential: at low concentrations (0.05 mg/l) GSPE induces depolarization in STC-1 cells, whereas at high concentrations (50 mg/l) it leads to hyperpolarization and the concomitant suppression of GLP-1 secretion [218]. In regard to carbohydrates digestion, PACs are able to inhibit some digestive enzymes even more than their anthocyanin relatives, suggesting excellent potential in suppressing the early glycemic spike and thus preventing T2DM [215,219,220,221]. For instance, proanthocyanidin B2 (PB_2_) reversibly and significantly inhibits α-glucosidase activity (IC_50_ = 0.23 ± 0.01 μg/mL), with only slight effect on α-amylase (IC_50_ = 0.86 mmol/L) on everted intestinal sleeves [185]. To elaborate—PB_2_ inhibited α-glucosidase in a mixed-type manner to interrupt the enzyme–substrate intermediate. Finally, molecular docking analysis revealed that PB_2_ interacts with several amino acid residues of α-glucosidase, thus inducing a conformational change, ultimately leading to aggregation [185]. PACs activity on digestive enzymes is strictly dependent on their structure: in particular, the number of hydroxyl groups, their position on the A, B, and C rings [222] and the degree of polymerization are critical [215,223]. Interestingly, Zhong and co-workers demonstrated that the PAC-mediated inhibition of some digestive enzymes in the small intestine and pancreas was more pronounced in mice fed high-degree PACs with respect to those fed low-degree PACs [215]. This effect is probably due to the presence of a higher number of phenolic hydroxyl groups in the high-polymer PACs, which may establish a larger number of hydrogen bonds with the peptidyl-NH-CO-, amino-NH_2_-, and carboxyl-COOH groups of α-amylases, thus forming a complex with reduced or lost catalytic capacity. Interestingly, the formation of enzyme aggregates may be affected by carbohydrates: pectin and Arabic gum cannot restore the enzyme activity but are able to reduce the formation of insoluble aggregates [224]. Similar results were also obtained measuring trypsin activity in the small intestine, which resulted inhibited by 32% in the high-polymer fed mice group and by 15% in the low-polymer group, and pepsin activity in the stomach inhibited at a rate of 38% and 13% by high- and low-degree PACs, respectively [215]. Conversely, lipase activity was not depressed by PACs, probably due to a lower affinity for this class of polyphenols than the other digestive enzymes [215]. Nevertheless, fat, as well as protein and mineral apparent digestibility is strongly affected by PACs supplementation, thanks to their great capability to interact with macromolecules and metal ions interfering with, and specifically hindering, their absorption and digestion. Furthermore, PACs may affect the discharge of nutrients interacting with polysaccharides, proteins, and phospholipids localized on cell membranes and thus conditioning their permeability. Moreover, in this case polymers showed the highest activity selectively dependent on their structure, molecular mass, and spatial configuration [215].

However, as previously said, 70% of the total PACs taken with the diet (mostly polymers) are not directly absorbed in the stomach and small intestine, but remain in the lumen of gastrointestinal tract and are massively metabolized in the colon before entering the systemic circulation in the form of metabolites [225]. In most cases, unabsorbed polyphenolic compounds can become substrates for fermentation of the fecal microbiota in the colon [226]. This is especially the case with polymeric PACs, which have revealed good potential against obesity-associated metabolic disorders altering gut microbiota profile. For instance, two very recent studies have shown that polyphenol-rich fractions purified from whole blueberry exert distinct effects on the fecal microbiota composition based on the type of bioactive compound [203,204]. In particular, PAC fractions revealed the greatest impact in promoting α diversity of the fecal microbiota specifically resulting in the most sustained content of Lachnospiraceae and Bacteroidaceae in an in vitro colon system [204]. Interestingly, Ntemiri and co-workers established a correlation between fecal microbiota changes and circulating antioxidant activity, showing that a subset of specific taxonomic groups enriched by blueberry consumption were also significantly and positively associated with ferric-reducing antioxidant power (FRAP), which, in turn, negatively correlated with the plasma glucose levels [204]. Moreover, the polymeric PAC-rich fraction leads to an improvement of glucose tolerance in vivo that has been strictly related to the modulation of bacterial taxa within the families Coriobacteriaceae and Verrucomicrobiaceae and the maintenance of the colonic mucus layer [203]. The latter is mainly due to an increase of the number of mucin-secreting goblet cells induced by polymeric PACs consumption [203,227]. Moreover, it seems that this protective effect on colonic mucus thickness could be mediated by an increase in acidic-mixed mucin secretion, more resistant against microbiological degradation than neutral mucins [203]. On the other hand, oligomeric PAC-rich fraction stimulates the abundance of bacteria known to play a key role in colonic epithelial immunomodulatory response and to protect against metabolic disorders like *A. muciniphila* [228,229,230], whose proportion significantly increases following PACs consumption [203,231,232,233]. Interestingly, this combines to lower urine content of metabolites associated with insulin resistance [233]. Another bacterium stimulated by PAC oligomers is *A. equolifaciens* [203], known to decrease concomitantly with inflammatory bowel disease development [234] and to be involved in the degradation of phenolic compounds including (−)-epigallocatechin, (−)-epicatechin, (−)-catechin, and (+)-catechin into their corresponding metabolites [235,236]. This evidence suggests a compelling involvement of PACs in their own metabolism, which is particularly relevant as it can generate bioactive molecules involved in the improvement of metabolic disorders. Finally, several human and animal studies have highlighted a correlation between metabolic disorders such as obesity and T2DM and a higher ratio Firmicutes/Bacteroidetes [237,238,239]. In this regard, GSPE and highly polymeric procyanidins impact on this ratio increasing Bacteroidetes and decreasing Firmicutes phyla [217,233].

Interestingly, PACs oligomers larger than decamer exhibited a strong absorption capacity of methyl mercaptan, hydrogen sulfide, and other putrefactive products both in vitro and in vivo [240]. The resulting strong deodorizing outcome of PACs on fecal odor may be due not only to the absorption of foul-smelling compounds from stool, but also by PAC-induced changes in the intestinal flora. In fact, proanthocyanidin-rich extract from grape seeds significantly enhances the number of *Bifidobacterium* and lowers *Enterobacteriaceae* in human fecal specimens [240].

#### 7.1.2. Liver: Glucose Uptake and Metabolism

Most PACs, upon absorption through the gut, travel from the portal bloodstream to the liver, where monomers undergo to phase I and II biotransformation through which they become more hydrophilic, thus favoring their entering the systemic circulation and secretion through the urinary system [241]. However, once in the liver, PACs oligomers may modulate hepatocytes functions and interfere with glucose uptake and metabolism.

For instance, PACs may reduce hyperglycemia through the regulation of GLUT2 transporters: in addition to stimulate basal glucose uptake into human HepG2 cells by significantly increasing GLUT2 expression (1.9–3.12-fold) [220], PACs can revert the decreased extracellular glucose consumption triggered by insulin pre-treatment, leading to an insulin sensitivity improvement similar to that observed upon treatment with metformin [242]. Furthermore, a correlation has been established between PAC exposure and/or supplementation with reduced gluconeogenesis and increased glycolytic and glycogenic activity in the liver, which ultimately results in lower levels of circulating blood sugar [220,242]. Indeed, PACs can inhibit the activity of hepatic glucokinase (GCK), a major player in glucose homeostasis responsible for converting glucose to glucose-6-phosphate [214,242] as well as increase the expression of a critical gene of glycogenesis, GYS2 [220]. PACs have also proved effective in preventing some secondary complications of long-standing hyperglycemia like glycation, a random process that occurs when macromolecules are found in an environment with a high concentration of sugars and impairs the functioning of biomolecules producing the so-called “advanced glycation end products” (AGE) responsible for many vascular complications in T2DM [220].

In addition to these effects, PACs also reduce hepatic glucose production. In particular, they dampen gluconeogenesis mainly through the activation of the adenosine monophosphate–activated protein kinase (AMPK) pathway. As demonstrated both in vitro and in vivo, PACs dose-dependently improve hyperglycemia and insulin sensitivity through the activation of the AMPK signaling pathway, which, in turn, lead to a significant hepatic downregulation of rate-limiting gluconeogenic enzymes, i.e., glucose-6-phosphatase (G6Pase) and phosphoenolpyruvate carboxykinase (PEPCK) [214,243,244,245]. Moreover, type 2 diabetic mice fed Enzogenol (EZ), a PAC-rich extract from the pine bark New Zealand *Pinus radiata* trees, showed a dose-dependent increase in the expression of hepatic glycogen synthase (GS), another key enzyme in glucose metabolism that is impaired in diabetes disorder [214]. However, AMPK activation is not the only mechanism through which PACs exert their glucose regulatory actions. Indeed, although results from an in vitro study on HepG2 showed that epigallocatechin gallate (EGCG) suppressed gluconeogenesis following ROS production and the subsequent calcium/calmodulin-dependent protein kinase (CaMKK)-mediated AMPK activation and not through the activation of the insulin signaling pathway [246], other pieces of evidence revealed that many PACs’ effects on hepatic glucose metabolism are mediated by the latter. For instance, Cordero-Herrera and co-workers demonstrated that EC from cocoa activated not only AMPK but also key proteins of the insulin pathways, including insulin receptor (IR) and insulin receptor substrate (IRS) 1 and 2, through the PI3K/Akt pathway both in vitro and in vivo [245,247,248]. The decrease in tyrosine-phosphorylated and total levels of IR, IRS-1 and -2, as well as PI3K/Akt pathway inhibition observed after high glucose exposure, was reverted after HepG2 pre-treatment with EC [247]. Similarly, in type 2 diabetic Zucker diabetic fatty (ZDF) rats fed a cocoa-rich diet (10%), hepatic insulin resistance is improved thanks to a reduced serine-phosphorylation of the IRS-1 and a strongly supported glycogen synthase kinase 3/glycogen synthase pathway [248]. Moreover, ZDF rats supplemented with cocoa showed significant suppression of events caused by insulin resistance such as c-Jun N-terminal protein kinase (JNK) and p38 activation [248]. These actions, together with GCK and GLUT2 improvement and PEPCK inhibition, give rise to the overall hypoglycemic effects shown by cocoa supplementation, resulting in reduced glucose and insulin levels in ZDF rats blood, as well as an improved glucose tolerance [248]. Consistently, in insulin-resistant Albino Wistar rats, a GSP diet (100 mg/kg) improves hyperglycemia and hyperinsulinemia, increasing tyrosine phosphorylation of IR-β and IRS-1 and decreased serine phosphorylation of IRS-1. Furthermore, the insulin signaling pathway is enhanced by GSP through the association between the PI3K p85α subunit and IRS-1 and the subsequent Akt phosphorylation [249]. Taken together, all of these findings explain the insulin-like effects shown by PACs and support their usefulness in countering what is one of the main issues associated with type 2 diabetes mellitus, namely insulin resistance.

#### 7.1.3. Pancreas: β-Cell Functionality

PACs, besides their inhibitory action on pancreatic digestive enzymes, such as α-amylase and trypsin [215,224], explicate their effects on glucose homeostasis also directly affecting major pancreatic β-cell functions, i.e., prevention of oxidative stress, enhancement of insulin secretion, and promotion of cell survival. The role of free radicals in the etiology of diabetes is well established [250]: starting from the well-known antioxidant properties of PACs, several studies focused on the role of this class of polyphenols in the control of OS in pancreatic β-cells. Islet cells are particularly susceptible to free radical attacks since their defense against ROS are weak. GSP administration attenuated some ER stress markers such as glucose-regulated protein (GRP) 78, C/-EBP homologous protein (CHOP) and caspase-12 and significantly decreased the activity of JNK, all dramatically elevated in pancreatic islets of diabetic rats [189]. PAC-mediated attenuation of ER stress in β-cells is also accompanied by an improvement in the pancreatic islet atrophy observed diabetic conditions and a reduction in the number of apoptotic cells both in vitro and in vivo [189,251,252]. Moreover, GSP oral administration has been shown to significantly reduce the pancreatic total nitrate/nitrite and ROS content and at the same time to increase pancreatic glutathione (GSH) levels, overall resulting in lower blood glucose and higher insulin levels in diabetic rats [188,251]. Therefore, carrying out their antioxidant activity, PACs promote β-cell viability, proliferation, and regeneration in a dose-dependent manner [253,254,255].

In addition to their protective effects against oxidative insults in pancreatic tissues, PACs directly stimulate insulin secretion, probably with greater efficiency the higher the number of hydroxyl groups on the B ring, as assessed for anthocyanins [256]. PACs promote glucose-stimulated insulin secretion (GSIS), both on healthy and diabetic rats [255,257], both on normal isolated Langerhans islets [255], and on β-cell lines [257,258]. This effect was further pronounced in rats fed with hydrolyzed PACs (HPAC), once again confirming that hydrolysis of the PAC polymers allows improving their benefits on glucose homeostasis and pancreatic β-cell function, by enhancing their bioavailability, as confirmed through the detection of increased PAC metabolites in serum samples from animals fed with HPAC [259]. PACs’ role as insulin secretagogues is probably due to their involvement as modulators of specific signaling pathways rather than their antioxidant properties. In fact, in contrast with studies on PACs as β-cell antioxidants, more than one finding showed that lower doses (≤15 mg/kg) of GSPE were more effective than higher doses (≥25 mg/kg) in promoting insulin secretion, especially in long-term treatments (≥1 month). Indeed, a prolonged high dose GSPE treatment decreased insulin production both ex vivo [192] and in vivo [192,260], and this was related to the downregulation of proteins involved in insulin synthesis and secretion like the pancreatic and duodenal homeobox 1 (PDX-1) [192,260]. PACs’ impact on insulinemia might be also attributable to a direct effect of PACs on insulin clearance: PACs affected mRNA expression of insulin degrading enzyme (Ide), responsible for the removal of insulin and particularly active in the liver [192]. However, the specific mechanism through which PACs may regulate Ide expression is still not completely clear. Taking into account the evidence that peroxisome proliferator-activated receptor-γ (PPARγ) plays an important role in regulating Ide gene expression in rat primary neurons [261], together with the finding that GSPE treatment downregulates PPARγ expression in 3T3-L1 adipocytes [262], PAC-mediated control of Ide expression could occur through PPARγ regulation.

#### 7.1.4. Insulin-Sensitive Tissues: Adipose Tissue and Muscle

PACs, thanks to their insulin-mimetic properties, influence glucose homeostasis even in insulin-sensitive tissues, such as adipose tissue and skeletal muscle. For example, as described for the intestine and liver, PACs stimulate glucose absorption in a dose-dependent manner also in adipocytes and myocytes, though different molecular mechanisms. They improve glucose uptake by upregulating GLUT4 expression [191,219,242,263], as well as promote GLUT4 translocation to the plasma membrane like insulin in both adipocytes and myocytes [191,243]. The PAC-mediated GLUT4 recruitment to the cell surface involves the activation of PI3K and p38 MAPK, as demonstrated by the sensitivity to both wortmannin and SB203580 [191]. In addition, pigmented rice bran extracts exerted a positive regulation of GLUT1 mRNA, which is essential for the biosynthesis of GLUT proteins to mediate the glucose uptake into adipocytes [219].

PACs, besides their effect on glucose transporters, increase the expression of genes encoding insulin-signaling pathway proteins, including Akt2, the isoform most strongly linked to GLUT4 translocation, PI3K and the insulin receptor substrate 1 (IRS1), which plays a key role in insulin-stimulated glucose uptake, in addition to the insulin receptor (IR) itself [219]. Procyanidin type-A oligomers, especially trimers and tetramers, increased IRβ levels in mouse 3T3-L1 adipocytes [263]. Montagut and co-workers demonstrated that PAC oligomers from GSPE are able to directly activate IR and other key targets of the insulin signaling pathway [264]. However, although PACs interact directly with the insulin receptor, the activation mechanism is slightly different from that triggered by insulin. More specifically, PACs phosphorylate Akt at residue Thr308 less than insulin but at Ser473 to a similar extent [264]. Moreover, PAC oligomers were found to phosphorylate p44/p42 and p38 MAPKs much more than insulin [264]. Finally, PACs ameliorate obesity and glucose intolerance by enhancing energy expenditure in adipose tissues. Black soybean seed coat extract consisting of 39.8% procyanidins leads to the up-regulation of uncoupling proteins (UCPs), whose role on energy metabolism and obesity has been largely investigated in the last three decades [265]. In particular, PACs enhance UCP-1 expression in brown adipose tissue, where it plays a key role in energy consumption by fat oxidation and following heat generation, and promote UCP-2 expression in white adipose tissue thus interfering with energy metabolism and obesity [207,266]. Through this action together with the removal of glucose from the bloodstream into adipocytes and myocytes and the promotion of the insulin signaling pathway, PACs may exert a significant protective activity against obesity, diabetes and other metabolic disorders by helping to improve glucose tolerance and homeostasis and reducing complications like insulin resistance.

### 7.2. Lipid-Lowering Effect

Hyperlipidemia, a condition characterized by disrupted lipid levels and dysregulated fatty acid and cholesterol metabolism, is associated with several pathological conditions such as obesity, MetS, coronary heart disease and atherosclerosis [3,5]. This condition is mainly defined by high serum concentration of low-density lipoprotein (LDL), low high-density lipoprotein (HDL) levels, hypertriglyceridemia and imbalanced redox homeostasis due to improved lipid peroxidation and increased LDL susceptibility to oxidation. Oligomeric PACs can act as cell signaling molecules to modulate lipid homeostasis in the systemic circulation, as revealed by numerous in vivo studies, which essentially indicate a PAC-related lowering effect on total cholesterol (TC), triglycerides (TG) or triacylglycerol (TAG), plasma free fatty acid (FFA) and LDL levels (Table 3).

**Figure 16:**
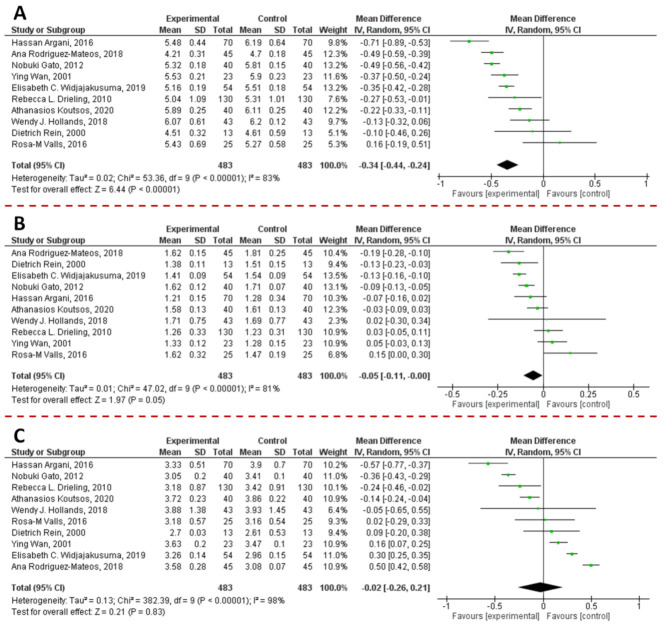
Forest plot representation of the effects derived from the supplementation of PACs on total cholesterol (**A**), HDL (**B**), and LDL (**C**) levels. Data were extrapolated from [187,267,268,269,270,271,272,273,274,275], and plotted according to the mean difference. Each horizontal line of the plot represents an individual study, reporting the punctual result plotted as green box. The weight of each study is represented by the size of the green box. The horizontal line indicates the lower and upper limit of the 95% Confidence Interval (CI) of the effect observed for each study. The vertical line represents the no-effect. For each study, if the horizontal line crosses the vertical one, a statistically significant difference between Experimental and Control group is not observed. The black diamond at the bottom of the forest plot represents the average effect size combining together the results of all the selected studies. The horizontal points of the diamond are the limits of the 95% CI of the average value. The figure was generated by Review Manager Software, version 5.4.1.

In order to understand if PAC supplementation could affect blood lipid levels, we performed a meta-analysis on data collected from articles published in the last 10 years and that satisfied the inclusion criteria established above. Briefly, the previously published articles (*n* = 248) were obtained by a literature search on PubMed, Scopus, Google Scholar, and ISI Web of Science research tool and using the following keywords: (“proanthocyanidin(s)” OR “procyanidin(s)” OR “PAC(s)” AND “cholesterol” OR “LDL” OR “HDL” OR “low density lipoprotein” OR “high density lipoprotein”). Then, a manual screening of the articles was performed by reading the title, abstract, or full text. Original articles were exclusively included if they met the following inclusion criteria: (i) the language should be English; (ii) articles should be published in peer-review journals; and (iii) after the reviewing by experts; (iv) the study design should be a randomized controlled clinical trials on humans; (v) the intervention should be the supplementation of formulation containing PACs only, and not in combination with other substances; (vi) only studies in which the number of participant have been clearly reported should be included; (vii) the parameters measured should be related to total cholesterol level, LDL, or HDL; (viii) when outcomes were presented at different times in the study, only the longest follow-up duration was selected. Accordingly, from the 248 published full text articles identified during the bibliographic search, 238 were excluded. Data from the selected articles (*n* = 10) were employed for the meta-analysis [187,267,268,269,270,271,272,273,274,275]. Since data were accumulated from a series of studies that had been independently performed, all of the selected studies were not functionally equivalent. Consequently, the originated forest plots (Figure 16) were performed using random effect, according to the heterogeneity calculated between the studies. Statistical heterogeneity among studies was checked with the Cochrane Q test (significance level of *p* < 0.05) and the I^2^ statistic.

Furthermore, sensitivity analyses were performed to evaluate the influence of each study on the overall effect size. Finally, potential publication bias was checked by visual inspection of the respective funnel plot. As the Figure 1 displays, no publication bias was identified among the selected studies for total cholesterol (Figure S1B) and HDL (Figure S1C) levels. However, it was observed on LDL levels (Figure S1D).

The combined results of the selected articles from the random-effect model suggested a significant effect of PAC supplementation on total cholesterol (WMD: −0.34 mmol/L; 95% CI: −0.44, −0.24; I^2^ = 83%; *p* = 0.00001) and HDL (WMD: −0.05 mmol/L; 95% CI: −0.11, −0.01; I^2^ = 81%; *p* = 0.05) levels. However, no effects were observed on LDL (WMD: −0.05, mmol/L; 98% CI: −0.26, +0.21; I^2^ = 98%; *p* = 0.83) levels.

**Figure 17:**
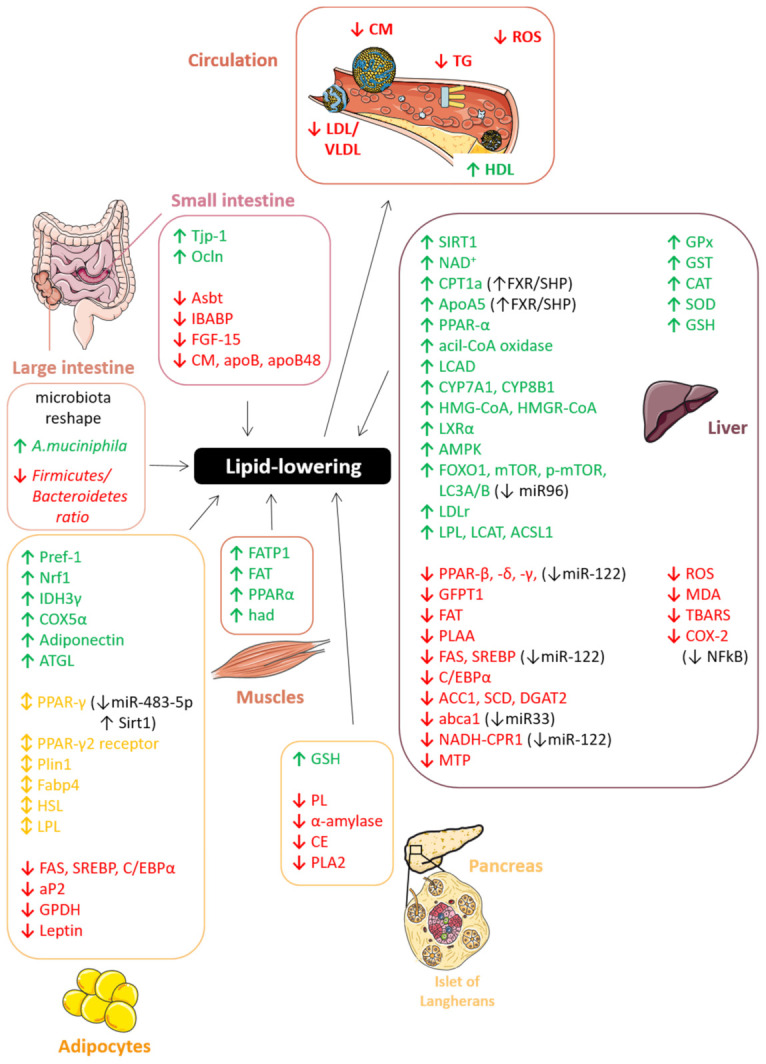
Schematic representation of the molecular mechanisms through which PACs affect lipid metabolism protecting against hyperlipidemia. ↑ increase; ↓ decrease; ↕ increase/decrease. The figure was created using Servier Medical Art by Servier (smart.servier.com accessed on 12 March 2021), licensed under a Creative Commons Attribution 3.0 Unported License.

In the next subsections, we will deepen the potential beneficial effects of PACs on hyperlipidemia. In particular, we will investigate the main molecular mechanisms through which PACs can interfere with lipid metabolism (Figure 17) which mainly concern intestinal absorption of lipids and the secretion of chylomicrons (CM) and lipoproteins by the intestine and liver [276].

**Table 3:** In vitro and in vivo studies on PAC-mediated and lipid-lowering effect.

Lipid-Lowering/Anti-Obesity Studies				
References		PAC Type or Source	Plasma Parameters	Model
Bansode et al., 2014	[277]	procyanidin A2	TG, VLDL	Rats
Yin et al., 2017	[278]	procyanidin B2	TG, TC, FFA	Mice
Xing et al., 2019	[279]	procyanidin B2	TG, TC, aspartate transaminase	Rabbits
Sano et al., 2007	[183]	PACs tablets	MDA-LDL, adiponectin	human
Mildner-Szkudlarz et al., 2013	[280]	grape seed and extract	TC, LDL-C, HDL-C, leptin, GLU	Rats
Natella et al., 2002	[281]	GSPE	TC, TAG, LPO, OS biomarkers	human
Del Bas et al., 2008	[282]	GSPE	TG, ApoB	Rats
Del Bas et al., 2009	[283]	GSPE	TG	Mice
Quesada et al., 2009	[284]	GSPE	TG, LDL-C	Rats
Adisakwattana et al., 2010	[285]	GSPE	TG, FC	Rats
Jiao et al., 2010	[286]	GSPE	TC, TAG	Hamsters
Pajuelo et al., 2011	[287]	GSPE	TG, FFA, glycerol, urea	Rats
Baselga-Escudero et al., 2013	[288]	GSPE	TG, TC, LDL-C	Rats
Guerrero et al., 2013	[289]	GSPE and GSPE metabolites	TG, FC, CE	Rats
Caimari et al., 2013	[290]	GSPE	FFA, PL	Hamsters
Hintz et al., 2014	[291]	GSPE	TG, BA	Mice
Downing et al., 2015	[292]	GSPE	TG, BA	Rats
Baselga-Escudero et al., 2015	[293]	GSPE	TG, LDL-C, HDL-C/LDL-C	Rats
Heidker et al., 2016	[294]	GSPE	TG, FC, BA, FFA	Mice
Shi et al., 2019	[295]	GSPE	TG, TC, LDL-C, HDL-C	Mice
Gonçalves et al., 2017	[296]	Vitis vinifera extract	LDL, adiponectin, leptin	human
Senault et al., 2000	[297]	red wine	HDL-C, Apo A-I, HDL3-C, LpA-I	human
Pal et al., 2004	[298]	red wine	ApoB48, CM, CMR, TC, LDL-C, HDL-C, TAG, GLU, INS	human
Sugiyama et al., 2007	[299]	apple	TG	Mice/ human
Rein et al., 2000	[275]	EC from chocolate	TG, TBARS, OS biomarkers	human
Wan et al., 2001	[274]	cocoa and dark chocolate	HDL-C, OS biomarkers	human
Mursu et al., 2004	[300]	chocolate	HDL-C, LDL diene conjugates	human
Mellor et al., 2010	[301]	chocolate	TC, HDL	human
Tokede et al., 2011	[302]	dark chocolate	TC, TG, LDL-C, HDL-C	human
Drieling et al., 2011	[273]	pine bark	LDL-C, GLU, INS, CRP	human
Yokozawa et al., 2008	[303]	Gravinol	TC, TG, LDL, VLDL, IDL, GLU, GP, TBARS	Rats

EC = epicatechin; GSPE = grape seed extract; GLU = glucose; INS = insulin; GLP-1 = glucagon-like peptide-1; TG = triglycerides; TC = total cholesterol; LDL -C = low density lipoprotein-cholesterol; ALT = alanine aminotransferase; HOMA-IR = homeostasis model assessment of insulin resistance; GP = glycosylated protein; NEFA = non-esterified fatty acids; OS = oxidative stress; HbA1c = glycosylated haemoglobin; BUN = blood urea nitrogen; HDL-C = high density lipoprotein-cholesterol; AST = aspartate aminotransferase; MDA = malondialdehyde; DAO = diamine oxidase; CRP = C-reactive protein; FRAP = ferric-reducing antioxidant power; GIP = glucose-dependent insulinotropic polypeptide; BA = bile acids; VLDL = very low density lipoprotein; FFA = free fatty acid; FC = free cholesterol; CE = cholesterol ester; PL = phospholipids; TAG = triacylglycerides; LPO = plasma lipid hydroperoxides; Apo = apolipoprotein; IDL = intermediate-density lipoprotein; TBARS = thiobarbituric acid-reactive substance; CM = chylomicrons; CMR = chylomicron remnants; LpA-I = lipoprotein particles A-I.

#### 7.2.1. Gut: Lipid Absorption and Chylomicron Secretion

The intestine is the first epithelium that dietary compounds encounter and it contributes to lipid homeostasis mainly by secreting triglyceride-rich lipoproteins during the postprandial state and by regulating lipid absorption. Long-chain fatty acids (LCFAs), free cholesterol, β-acyl glycerol, and bile salts form mixed micelles in enterocytes, whereas glycerol and short-/medium-chain fatty acids are directly absorbed from the intestinal lumen into the portal vein and will be further metabolized into the liver. PACs were found to reduce cholesterol and bile acid (BA) absorption by decreasing the micellar solubility of cholesterol [304,305]. It is thought that GSPE binds to BA, such as taurocholic acid, taurodeoxycholic acid, and glycodeoxycholic acid, forming insoluble complexes in the intestine, which inhibit the formation of cholesterol micelles in the intestinal lumen and increase their fecal excretion [285,305]. Moreover, procyanidin A1, and even more epicatechin-(4β→6)-epicatechin-(2β→O→7, 4β→8)-catechin extracted from the skin of the peanut, degrade cholesterol micelles in vitro, thus contributing to the overall cholesterol-lowering effect observed in rats fed with high-cholesterol diet supplemented with peanut skin [306]. Interestingly, apple highly polymeric procyanidins may mitigate intestinal permeability through the upregulation of tight junction protein-1 (Tjp1) and occludin (Ocln) in mice fed with high-fat/high-sucrose diet [233]; on the other hand, GSPE reduces intestinal apical sodium-dependent bile acid transporter (Asbt) [291,294] and intestinal bile acid-binding protein (IBABP) expression, thereby impacting BA uptake and transport through the enterocyte [291]. BA synthesis is also impacted by GSPE administration, as revealed by the repression of the ileal fibroblast growth factor 15 (FGF-15), a critical gut-liver regulator of cholesterol-7α-hydroxylase (CYP7A1), which is a key rate-limiting enzyme involved in BA synthesis [291].

Once in the intestinal epithelial cells, TGs are packed together with cholesterol and fat-soluble vitamins into CMs. This class of ultra-low-density lipoproteins is responsible for the transport of dietary lipids from the gut to other locations in the body within the water-based solution of the bloodstream. GSPE assumption affects postprandial hypertriacylglycerolemia by CM and/or VLDL regulation in a time-dependent manner. To elaborate, GSPE was shown to restore rat plasma TAG levels upon their high-fat diet (HFD)-induced increase [307]. Concomitantly, VLDL-TAG and CM-TAG levels in the plasma were reduced: after 1h the VLDL-rich fraction was the major contributor, while after 3 h the CM-rich fraction was prevalent (85%) [307]. However, the lipoprotein decrease mediated by PACs is not due to an increase in lipoprotein lipase (LPL) activity, but rather to repression of lipoprotein secretion [307]. Indeed, PACs reduce CM secretion, delaying the absorption of triglyceride and cholesterol in the intestine. More specifically, apple polyphenol extracts rich in PACs prevent the cholesteryl ester synthesis and lipoprotein secretion by human Caco-2/TC7 enterocytes. The latter is mainly due to the inhibition of ApoB synthesis, a marker of intestinal CM, rather than to its degradation [308]. Similarly, it has been shown that in Caco-2 cells incubated with red wine the intracellular cholesterol availability is brought down as well as CM synthesis and secretion since the expression of apolipoprotein B48 (ApoB48) was significantly reduced in CaCo-2 cells [309]. This result was also confirmed in a clinical study performed on dyslipidemic postmenopausal women, showing that the red wine assumption for 2 weeks induces a decrease in postprandial ApoB48 levels, indicating a delay in the absorption of dietary fat through a reduction in CM and CM remnant plasma levels [298].

Another mechanism through which PACs protect against hyperlipidemia-associated disorders is the regulation of the intestinal microbiota, as already described in the previous paragraph on hypoglycemic action. In particular, the reduction of hypertriglyceridemia plasma markers, such as triglycerides and total cholesterol, correlates with a significant improvement in the proportions of *Bacteroidetes* at the phylum level and *Akkermansia muciniphila* at the genus level in rabbits fed with procyanidin B2 [279]. Likewise, highly polymeric procyanidins increase the proportion of *A. muciniphila* by eight times in a mouse model fed a with high-fat/high-sucrose diet [233]. At the same time, they significantly decrease the Firmicutes/Bacteroidetes ratio and this change is accompanied by a reduction in butyrate, an energy source for colonocytes, and modulation of the ratio of acetate/propionate/butyrate [217]. The microbiota and the subsequent microbiota-derived short-chain fatty acids (SCFAs) reshaping evoked by PACs might be another mechanism through which this class of polyphenols protect against metabolic disorders, causing a reduction in plasma TAG and adiposity. Finally, as revealed by 16S rDNA analyses, *Clostridium* XIVa, *Roseburia,* and *Prevotella* are substantially modulated by GSPE supplementation on mice fed with HFD. Moreover, gut microbiota depletion by antibiotic treatment abolished some beneficial effects of GSPE, i.e., the reduction of the epididymal fat mass, further confirming the close microbiota-PAC activity relationship [310].

#### 7.2.2. Liver: Lipogenesis, Cholesterol Metabolism and LDL Secretion

The liver is probably the main organ in which PACs modulate lipid metabolism. Through a metabolomics approach PACs were shown to affect hepatic metabolism, mainly dose-dependently increasing its content in nicotinamide adenine dinucleotide (NAD^+^) [311]. The modulation of NAD^+^ precursors and the regulation of the expression of genes involved in its metabolism together with the upregulation of Sirtuin 1 (Sirt1) raises the hepatic activation of SIRT1 and thus reduces TG accumulation in the liver [311]. Moreover, pine bark extracts (Flavangenol) revealed a good potential in the treatment of NAFLD and NASH blocking hepatic fat accumulation both in vivo and in vitro [312]. Among all the components of Flavangenol, procyanidin B1 specifically promotes the oxidation of free fatty acids (FFAs) and regulates the expression of fatty acid oxidative enzymes such as acyl-CoA oxidase and carnitine palmitoyltransferase (CPT1) [312]. Similarly, another pine bark extract named Enzogenol enhanced long-chain acyl-CoA dehydrogenase (LCAD) protein level in db/db mice [214]. The expression of genes regulating lipid uptake, such as the proliferator-activated peroxisomal receptor γ (PPAR-γ), was downregulated, whereas PPAR-α, which leads to decreased TG and cholesterol levels in plasma and liver, was upregulated by PACs [208,214,313,314].

PACs are also actively involved in the suppression of hepatic lipogenesis, leading to reduced cholesterol, TG and FFA levels in a dose-dependent manner [278,303,314]. Liver proteome analysis on rats suffering from MetS revealed 75 proteins showing a differential expression in rats fed with HFD and supplemented with GSPE with respect to the control [315]. More specifically, GSPE downregulates genes involved in hepatic lipogenesis such as glutamine-fructose-6-phosphate transaminase 1 (GFPT1), fatty acid translocase (FAT) and phospholipase A2-activating protein (PLAA) [315]. PACs from cocoa, French maritime pine bark and grape seed were evaluated for their effects on lipid homeostasis evidencing that HepG2 cells treated with sera collected from rats administered PACs display a significant decrease in the de novo lipid synthesis [289]. The reduction observed on cells treated with GSPE rat serum metabolites was significantly higher than that induced by the direct treatment with GSPE extract, supporting the key role of PACs metabolism/conjugation in the expression of their pharmacological activity [289]. The PAC-mediated inhibition of hepatic lipogenesis arise from the downregulation of enzymes involved in the fatty acid synthesis, i.e., fatty acid synthase (FAS), sterol regulatory element-binding protein (SREBP)1 and 2, CCAAT-enhancer-binding proteins (C/EBP-α), acetyl-CoA carboxylase (ACC1), AMP-activated protein kinase (AMPK), carnitine palmitoyltransferase-1a (CPT-1a), and stearoyl-CoA desaturase 1 (SCD) [208,233,279,292,313,314]. More specifically, FAS and C/EBP-α modulation appear to be mediated by the inhibition of the c-Jun N-terminal kinase (JNK) signaling pathway induced by GSPE [316]. These regulatory effects on gene expression are primarily dependent on oligomeric rather than polymeric PACs [208,279,314]. One of the molecular mechanisms underlying the anti-lipogenic activity of PACs follows the same pathway as bile acids which involves the activation of the farnesoid X receptor (FXR) and the nuclear receptor small heterodimer partner (NR0B2/SHP) [282,294]. More specifically, PACs, as well as BA, are able to bind to and activate FXR, thus inducing the expression of its target SHP, which, in turn, regulates the expression of several lipogenic genes including SREBP-1c, CPT-1a and apolipoprotein A5 (ApoA5). FXR or SHP suppression via siRNA or knockout completely abolished the TG-lowering action of GSPE both in vitro and in vivo, confirming their essential role as mediators of the hypotriglyceridemic actions of PACs [282,283]. Moreover, a possible interaction of PACs with the transcription factor EB (TFEB) was also highlighted. Procyanidin B2, probably through direct interaction with TFEB, modulates its activity and consequently the expression of its target genes (Lamp1, Mcoln, Uvrag) involved in the lysosomal pathway in HFD-induced liver steatosis [314]. These results could identify procyanidin B2 as a promising candidate for the prevention and treatment of NAFLD.

PAC hypolipidemic effect is further enhanced thanks to an active role in microRNA regulation (miR). In particular, PACs were shown to rapidly and transiently repress miR-33, which targets ATP-binding cassette A1 (abca1) and genes involved in the modulation of fatty acid and cholesterol homeostasis, and miR-122, which targets fatty acid synthesis genes (e.g., srebp-1c, fas) and fatty acid β-oxidation genes (e.g., NADPH-cytochrome P450 reductase-1, ppar-β/δ) [288,293,317,318]. Their deregulation has been associated with metabolic disorders like obesity and MetS. A correlation of miR-33 and miR-122 levels with lipemia in nutritional rat models, hepatic and peripheral blood mononuclear cell lines (PBMCs) has been established [288]. Their sustained overexpression in dyslipidemia conditions is neutralized by long-term supplementation with GSPE [288,293]. In addition, GSPE attenuates the high-fat diet-induced overexpression of miR-96 and, consequently, of its downstream molecules such as FOXO1, mTOR, p-mTOR, and LC3A/B, which are known for improving the autophagic flux for clearance of lipid accumulation [295]. Going into more detail on the molecules responsible for this miR inhibition, it has been shown that A-type ECG and EGCG dimers specifically ameliorate hepatic steatosis significantly reducing lipid accumulation in L02 cells through the regulation of miR-122 and miR-33b and their target genes [318]. However, the exact molecular mechanism by which PACs may affect miRs regulation has not yet been fully elucidated: it could underlie the binding to components involved in miRs biogenesis or, alternatively, the direct binding of PACs to miRs to modulate their stability or degradation.

Other signaling pathways influenced by PACs involve cholesterol metabolism and catabolism. Under high fat intake, procyanidin B2 from *Annurca* apples reduces cholesterol synthesis by diverting citrate and acetyl-CoA to the Krebs cycle [319]. Concomitantly, it lowers fatty acid synthesis and promotes lipolysis and fatty acid β-oxidation thanks to a boost in mitochondrial activity [319]. Moreover, improved cholesterol degradation and excretion contribute to the cholesterol-lowering effect of PACs: GSPE causes a significant decrease in plasma TC and TAG levels in hamsters and rats fed with HFD [286,292]. This effect is mediated by the overexpression of CYP7A1 at both the transcriptional and protein levels [286,294]. Moreover, GSPE decreases serum BA levels improving its fecal excretion [292], as revealed by the upregulation of 3-hydroxy-3-methylglutaryl coenzyme A (HMG-CoA) and HMG-CoA reductase (HMGR-CoA) [233,286,307,320] and liver X receptor alpha (LXRα) mRNA [286]. These results, together with those previously described in enterocytes, support the hypothesis that PACs, acting as intestinal gene selective bile acid receptor modulators (BARM), and contribute to the TG-lowering by altering enterohepatic BA recirculation. Interestingly, co-administration of GSPE with the BA sequestrant cholestyramine (CHY) has been shown to be a valid lipid-lowering combination therapy, effective in further attenuating dyslipidemia by reducing hepatic cholesterol synthesis, improving BA biosynthesis and decreasing lipogenesis in mice [294].

PACs’ effects on postprandial hypertriacylglycerolemia are also due to the repression of lipoprotein secretion [284,307]. Indeed, oligomeric PACs increase low-density lipoprotein receptor (LDLr) expression and increase the activity of hepatic LPL, lecithin–cholesterol acyltransferase (LCAT) and serum paraoxonase and arylesterase (PON)-1, which associate with HDL in the circulation [320]. PAC treatment significantly reduces the secretion of VLDL-TAG and affects the hepatic expression of ACSL1 (acyl-coenzyme A synthetase long-chain family member 1), Apoc3, ApoA5, ApoB, HMG-CoA, HMGR-CoA, MTP (microsomal triglyceride transfer protein), DGAT2 (diacylglycerol O-acyltransferase 2) and the activity of CPT1a in high-fat/high sucrose conditions [233,282,284,307,320]. Differently from what was observed concerning CPT-1a and ApoA5 expression, the inhibition of ApoB secretion in HepG2 seems to be SHP-independent [282]. However, to the best knowledge of the authors, the exact molecular mechanism underlying this inhibition is still unclear.

In the next section, we will explore, in more detail, the mechanisms by which PACs affect blood lipoprotein levels.

#### 7.2.3. Pancreas: Lipid Degradation and β-Cell Functionality

Several pieces of evidence indicate that PACs inhibit pancreatic enzymes involved in lipid metabolism, including lipase, α-amylase, phospholipase A2 and cholesterol esterase. In general, this outcome is maximal with pentamer or greater procyanidins, whereas catechins and epicatechins didn’t show any activity [299]. PACs from grape seed and cocoa dose-dependently decrease the activity of pancreatic lipase (PL) (IC_50_ = 3.71 ± 0.03 mg/mL) in vitro [285,321,322]. PACs interact with porcine PL inducing and stabilizing aggregate formation; the resulting effect is a non-competitive dose-dependent inhibition of PL activity without variations in the K_m_ value while V_max_ decreases due to a reduction in the α-helix content and an increase in β-sheets [323,324]. The same effect was observed on pancreatic α-amylase and also in this case is due to the formation of enzyme aggregates [224,322,324]. Finally, PACs reduce cholesterol esterase activity (IC_50_ = 27.27 ± 4.12 mg/mL) [285,305] and inhibit secreted phospholipase A2 (PLA2) in a non-competitive manner [324].

Thanks to their lipid-lowering effect, PACs also revealed a protective effect on pancreatic β-cell functionality [325]. GSPE administration lowers TG content both in vitro and in vivo, consistently with the down-regulation of insulin and Pdx1 observed in pancreas and that of lipid synthesis-related genes like Fasn and Srebf1 in liver [325]. PACs’ inhibitory effect on TG accumulation in β-cells may explain their positive action on glucose homeostasis maintaining healthy levels of insulin production also under hyperlipidemic conditions.

#### 7.2.4. Adipose Tissue: Adipogenesis, Lipolysis, and Adipocytes Differentiation

White adipose tissue (WAT) represents the organism’s largest energy reservoir in mammals. Its anomalous expansion is closely related to insulin resistance and dyslipidemia and thus to obesity-related metabolic complications. In particular, in pathological conditions, WAT expands mainly through an increase in the size of the adipocytes (hypertrophy) and instead shows an impaired proliferation (hyperplasia). GSPE administration in rats fed with an obesogenic diet promotes a healthier expansion of retroperitoneal WAT (rWAT), by increasing the number of adipocytes and decreasing the adipocyte size in a dose-dependent manner [326,327]. At the molecular level, this effect mainly arises from the PAC-triggered induction of the expression of key lipolytic transcription factors, such as hormone-sensitive lipase (HSL), adipose triglyceride lipase (ATGL), and LPL and the enhanced release of both free fatty acid and glycerol [321,328]. Moreover, GSPE prevents lipid and TG accumulation in WAT, by upregulating the expression of genes involved in β-oxidation and glycerolipid/free fatty acid (GL/FFA) cycle and improving heparin-releasable LPL activity primarily in retroperitoneal and mesenteric WAT [290]. Furthermore, GSPE enhances adipogenesis in mature adipocytes through a Sirt1-dependent upregulation of PPAR-γ, which is its master regulator [327] and it improves perilipin 1 (Plin1), fatty acid–binding protein 4 (fabp4), and adiponectin expression thus ameliorating the overall WAT activity [326]. An interesting study by Ardevol and collaborators then showed that the PAC-mediated protective effects on fat deposits strongly depend on the metabolic conditions of the animal model: in Wistar lean rats PACs target mesenteric white adipose tissue (mWAT), whereas in Zucker obese rats they mainly target subcutaneous white adipose tissue (sWAT) [329]. However, whatever adipose tissue is affected, GSPE alters gene expression, but not in adiposity parameters [329]. Nevertheless, another study showed how hamsters fed with an HFD and supplemented with GSPE for 15 days displayed beneficial effects on body weight and fat accumulation: GSPE significantly lowers the adiposity index and the weight of all the white adipose tissue depots studied, i.e., rWAT, mWAT, eWAT, and inguinal (iWAT) [290].

Brown adipose tissue (BAT) exerts an anti-obesity effect, consuming energy via heat production. Moreover, it plays an immunometabolic role in the development of obesity, as revealed by the strong attenuation of the obesity-associated inflammatory markers in epididymal white adipose tissue (eWAT) induced by BAT transplantation in obese mice [330]. Obesity impairs BAT mitochondrial function and thermogenic capacity and GSPE can revert these dysfunctions improving the expression of Sirt1, nuclear respiratory factor 1 (Nrf1), isocitrate dehydrogenase 3γ (IDH3γ), and COX5α, and, more interestingly, the levels of mitochondrial respiration with both pyruvate and carnitine-palmitoyl-CoA as substrates [287,331]. In addition, it has been recently suggested that PACs might induce WAT browning [332]. However, to date, there is still no evidence of this potential functionality.

PACs have also been related to adipocyte differentiation, showing that GSPE can interfere with the early stages of 3T3-L1 (preadipocyte) differentiation into adipocytes. In particular, GSPE treatment inhibits pre-adipocyte differentiation decreasing the expression of the PPAR-γ2 receptor, which is the main regulator of adipocyte differentiation [262]. Accordingly, at the onset of differentiation adipose-specific markers were reduced, whereas pre-adipocyte factor-1 (pref-1) levels were maintained high by GSPE treatment [262,328]. In general, PACs drop lipid accumulation during the early stages of 3T3-L1 differentiation inhibiting both adipogenesis and lipolysis. Indeed, GSPE was shown to downregulate the expression of key regulators of lipid synthesis like PPAR-γ, C/EBP-α, SREBP1, FAS, PLIN1, FABP4, and adipocyte fatty acid-binding protein (aP2) [333,334]. This transcriptional regulation is probably mediated by the PPAR-γ signaling pathway, since GSPE treatment also reduced the expression of several genes involved in that pathway, including Adipoq, Scd1, Nr1h3, Fabp5, Scd2, and PPAR-γ itself in 3T3-L1 [333,335]. Moreover, PACs from lyophilized cranberries showed an inhibitory effect against lipolytic enzymes including LPL, HSL, and glycerol-3-phosphate dehydrogenase (GPDH) [328,335]. As previously described for the liver, PACs reduce intracellular lipid accumulation in adipose tissue also through the regulation of miRs. In particular, procyanidin B2 from grape seed was shown to impair adipogenesis and adipogenic differentiation in 3T3-L1 cells by repressing miR-483-5p and, thus, leading to lower activation of PPAR-γ [336]. Furthermore, PACs inhibit pre-adipocyte proliferation, as revealed by the downregulation of genes involved in the cell cycle and growth, the cell cycle arrest at the G_0_/G_1_ transition phase and the cell apoptosis observed following GSPE treatment on 3T3-L1 cells [262,328]. Finally, PACs dose-dependently increase adiponectin expression and decrease leptin levels, thus interfering with blood glucose levels as well as fatty acid breakdown [335]. The occurrence of obesity is closely related, among others, to the secretion of adipokines by adipose tissue [337]. Indeed, adipokines contribute to peripheral insulin resistance and disorders of lipid metabolism mainly interfering with insulin signaling pathways. In this regard, GSPE’s positive effect on adipokine secretion and oxidative stress validates their potential in fighting obesity and metabolic disorders [296,335,338].

As for the impact of PAC intake on the metabolic profile, it has even been shown that this goes beyond the individual to even affect the progeny [339,340,341]. GSPE administration during pregnancy and lactation might program offspring toward improved metabolism in adulthood. As an example, chicks at hatching and 10 days of age revealed increased live body weight and greater viability associated with a decrease in plasma and liver oxidative stress [338]. Moreover, it has been shown that in the offspring of rats that were fed with an HFD and that were treated with GSPE the expression of 238 eWAT genes was altered mostly toward a better inflammatory profile and an enhanced lipidic and glucosidic metabolic profile [340]. However, also deleterious programming effects on offspring have been reported, raising concerns about the possibility of using GSPE as a nutraceutical supplement during pregnancy. Indeed, adult male offspring of rats assuming GSPE during lactation display increased insulin resistance and impaired adiponectin pathway probably due to the higher lipid transfer to the pups through the milk following the GSPE-induced increase in the expression of lipogenic genes in the mother’s mammary glands [341].

#### 7.2.5. Skeletal Muscle

The role played by the skeletal muscle is well-known in the overall utilization and oxidation of fatty acids, and PACs also affect energetic metabolism in this tissue. Indeed, GSPE improves pyruvate consumption as a substrate for mitochondrial energy production and downregulates the mRNA expression of the fatty acid transport protein 1 (FATP1); thus, promoting glucose metabolism and reducing the insulin-sensitive cellular uptake of LCFAs [287]. Consequently, postprandial serum LCFA levels are altered and lipids are redistributed from adipocyte tissue and muscle to the liver, thereby reducing obesity-related metabolic complications.

Moreover, healthy rat male offspring supplementation with GSPE reduces blood C-reactive protein levels, improves lipid oxidation and AMPK expression and activity in the skeletal muscle. Similarly, the expression of genes involved in fatty acid uptake (Fatp1 and fat) and β-oxidation (PPAR-α and had) are overexpressed in their muscle upon GSPE administration [339].

#### 7.2.6. Plasma OS and Lipoproteins

The deregulation of lipid metabolism is closely linked to oxidative stress. Oligomeric PACs significantly affect the LDL/HDL ratio thanks to their antioxidant properties and to their capability to reduce lipid peroxidation. The latter not only depends on their bioavailability, but also on their ability to bind LDL and VLDL [342]. Many in vitro and in vivo studies have demonstrated that PAC assumption significantly improves plasma total antioxidant capacity and decreases plasma lipoprotein oxidation [343,344], by reducing, as an example, the copper-catalyzed oxidation of LDL [345], plasma 2-thiobarbituric acid reactive substances level (TBARS) and the expression of nuclear factor kappa B (NF-κB) and cyclooxygenase-2 [208,275,303]. Several clinical evidences on healthy volunteers support the PAC-mediated activity on postprandial OS in plasma. For instance, GSPE and red wine assumption not only decreases the content of plasma lipid hydroperoxides (LPO) and malondialdehyde-modified LDL (MDA-LDL) in healthy humans within the postprandial phase [183], but seems also to make LDL less susceptible to oxidative modification [281,346]. Consistently, cocoa powder and dark chocolate supplementation leads to an increase in LDL oxidation lag time, lower serum LDL diene conjugates (used as a marker of lipid peroxidation in vivo) and higher HDL-C plasma levels [274,300]. In addition, a clinical trial performed on 56 healthy young men showed that red wine significantly increased serum HDL-C, HDL3-C, Apo A-I, LpA-I, and LpA-I/LpA-II particles [297]. It should be noted that the HDL containing apo A-I but no apo A-II (LpA-I) can promote cholesterol efflux from cells, thus exerting a protective effect through the reverse cholesterol transport, while HDL containing apo A-I and apo A-II cannot. Then, not by chance, HDL-C, HDL3-C and HDL-phospholipid variations were found to positively correlate with serum-promoted cellular cholesterol efflux from hepatic Fu5AH cells treated with red wine [297]. This finding suggests a double effect of red wine on lipid homeostasis: on one hand, it influences lipoprotein-mediated cholesterol transport in the bloodstream, and on the other hand it gains serum-dependent efflux of cellular cholesterol. The ability of PACs and (+)-catechin from red wine to mainly bind to Apo A-I in humans and transferrin in rats further corroborates an involvement of PACs in reverting cholesterol transport [347]. Going deeper into the molecular details of PACs action it has been observed that they affect ROS, glutathione (GSH), and MDA intracellular levels [208,314]. Oligomers lower the generation of ROS and lipid peroxidation and improve the reduced glutathione/oxidized glutathione ratio [208]. Moreover, PACs can modulate the activity of many critical antioxidant enzymes including glutathione peroxidase (GPx), glutathione S-transferase (GST), catalase (CAT), and superoxide dismutase (SOD) [314,348]. In this context, EGCG treatment promotes Nfr2 nuclear accumulation and transcriptional activity [349]. This action comes from the activation of the Akt and ERK1/2 signaling pathways and leads to the modulation of the antioxidant response element (ARE)-mediated expression of many antioxidants as well as detoxifying enzymes. These activities, together with the restoration of lipid regulatory enzyme-like 5’ adenosine monophosphate-activated protein kinase (AMPK) and ACC phosphorylation [278], lead to an improvement in lipid peroxidation damage ultimately resulting in serum LDL/HDL ratio lowering.

### 7.3. Intestinal Inflammation

Intestinal inflammatory diseases are contemporary conditions of industrialized societies. Their increased incidence has been associated with the westernization of diet and environment, with strong changes in intestinal microbiota, and with continuous intestinal epithelial cell exposure to pesticides, food additives, drugs, and other food chemicals [350,351,352]. To date, adequate strategies for the prevention or treatment of inflammatory gut diseases are still lacking. Several studies have evaluated the influence of dietary components in the prevention and treatment of intestinal inflammation and protective effects of several polyphenols were reported [165]. In particular, increasing data from in vitro and in vivo studies showed protective effects of proanthocyanidins on intestinal epithelium supporting positive effects of PACs and PAC rich-foods for the physiology of the gastrointestinal tract. The main manuscripts describing the anti-inflammatory potential derived from the intake of PACs are reported in Table 4 and Table 5.

Several in vivo studies (Table 5), using murine models of experimental colitis, showed that PACs have anti-inflammatory effects in intestinal bowel diseases (IBD). Oral administration of PAC-rich extracts leads to significant protection against epithelial barrier dysfunctions [353,354,355], mainly exerted through the inhibition of TNF-α, INF-γ, and IL-1β release, reduced myeloperoxidase activity [310,355,356,357], inhibition of NF-κB signaling pathway [358,359,360], and increased antioxidant enzymes (GPx and SOD) activity [361]. Despite these studies revealing a potential beneficial role of PACs in intestinal inflammation, the mechanisms involved in this protective effect have not yet been fully clarified. One of the mechanisms involved undoubtedly concerns the antioxidant properties of PACs: Wu et al. showed that incubation of intestinal epithelium with proanthocyanidin dimers prevented LPS-mediated oxidative stress increasing SOD, HO-1, CAT, and GSH-Px mRNA expression [362]. Furthermore, the ability of PACs to preserve the barrier function of intestinal epithelial cells mentioned above [165,363] contributes to increasing paracellular permeability in inflammatory conditions, for example, through the overexpression of tight junction proteins [362]. However, intestinal inflammation is a complex process that involves different cell types in the gut. Although activation of mucosal inflammatory cells is important in in vivo inflammatory response, intestinal epithelial cells also play a crucial role by actively releasing inflammatory mediators and modulating the intestinal permeability. Monolayers of differentiated Caco2 cell line stimulated with pro-inflammatory agents and then treated with PACs showed a decrease in the release of pro-inflammatory cytokines (TNF-α, IL-6, IL-8), cyclooxygenase (COX)-2 expression, prostaglandin E2 production, and a reduced activation of NF-κB [163,165,362,364].

**Table 4:** In vitro studies reporting the anti-inflammatory activity of PACs.

Extracts	Concentration	Pro-Inflammatory Inductors	Results	Ref.
Cranberry proanthocyanidins extract	250 µg/mL	Fe/Asc mixture and LPS	↓ PGE2, ↓ COX-2, ↓ TNF-α, ↓ IL-6	[364]
Pistachio nut proanthocyanidins extract	4.8–12 mg CE/ml	IL-1β	↓ IL-6, ↓ IL-8, ↓ (PG)E2, ↓ COX2, ↓ Iκ-Bα phosphorylation, ↓ FSA permeation, ↑ TEER,	[165]
Hexameric procyanidins from GSE	20 µM	TNF-α	↓ NF-κB activation, ↓ ROS	[163]
Granny Smith apple procyanidin extract	12.5–100 μg/mL	LPS	↑ (ZO)-1, ↑ SOD, ↑ HO-1, ↑ CAT, ↑ GSH-Px, ↓ NF-κβ, ↓ IL-6, ↓ TNF-α	[362]
Cocoa procyanidin polymers	100 µg/mL	DSS (Caco2 cell line) and TNF-α (HT29 cell line)	↓IL-8	[363]

**↑** increase; **↓** decrease.

**Table 5:** In vivo studies reporting the anti-inflammatory activity of PACs.

Extracts	Dose	Pro-Inflammatory Inductors	Results	Ref.
Grape seed proanthocyanidin extract	5, 25, or 50 mg/kg body weight	Cafeteria diet	↓ IL-1β, ↓ iNOS, ↓ MPO activity, ↓ ROS, ↑ ZO-1	[354]
Grape seed proanthocyanidin extract	75 or 375 mg/kg body weight	LPS	↓ COX-2 activity, ↓MPO activity, ↓ ROS, ↓ Plasma OVA	[355]
*Pyracantha fortuneana* fruit extract	0.4 or 1 g/100 g of dry feed weight	High-fat diet	↑ Occludin, ↑ ZO-1	[353]
Grape seed proanthocyanidin extract	0.1 g/100 mL of drinking water	(Colitis in IL-10 deficient rats)	↓ TNF-α, ↓ IFN-γ, ↑ iNOS	[356]
Grape seed proanthocyanidin extract	300 mg/kg body weight	High-fat diet	↓ TNF-α, ↓ IL-6, ↓ MCP-1	[310]
Procyanidin B2	10, 20, or 40 mg/Kg	DSS	↓ MMP9, ↓ TNF-α, ↓ IL-1β, ↓ IL-6	[360]
Grape seed proanthocyanidin extract	100, 200, and 400 mg/kg	TNBS	↓ NF-Κb, ↓ pIκBα, ↓ IκK	[359]
Grape seed proanthocyanidin extract	25 and 50 mg/kg	Cafeteria diet	↓ TNF-α, ↓ IL-6, ↓ NF-Κb, ↓ Emr1, ↓ CPR	[358]
Grape seed proanthocyanidin extract	100, 200, and 400 mg/kg	TNBS	↑ GSH-Px, ↑SOD, ↓ TNF-α, ↓ p-IKKα/β, ↓ NF-κb	[361]

↑ increase; ↓ decrease.

Literature data demonstrating protective effects of PAC-rich extracts in intestinal inflammation tend to attribute the observed effects to the high molecular weight proanthocyanidin fraction [163,165]. Furthermore, the demonstrated activity of these extracts in in vitro models of intestinal epithelium would suggest protective actions of physiological relevance. In fact, high molecular weight PACs, thanks to their stability under gastrointestinal digestion conditions and their reduced ability to cross biological membranes, can reach significant concentrations in the intestinal lumen. In this context, the anti-inflammatory proprieties of large PACs could involve events initiated at the level of cell membranes with modulation of proteins at key sites of different signaling pathways [365]. In this regard, several studies have demonstrated the interaction of large proanthocyanidin with areas of cell membrane with reduced mobility, such as lipid rafts [366,367] and its central role in mediating the anti-inflammatory activity shown by polymeric PACs [163].

## 8. Conclusions

PACs are polyphenolic compounds that have been shown to have an interesting and wide range of bioactivities. The information contained in this review clarifies the particular chemical characteristics of PACs, explaining their highly variable chemical scaffold. Furthermore, in this review, the biosynthetic pathway was deeply discussed, also highlighting the main shortcomings of this processes. In particular, the potential mechanisms of transport and polymerization of PACs in plant cells was hypothesized. From an analytic point of view, the main protocols aimed at identifying and quantifying PACs in plant sources have been described, also emphasizing the main advantages and limitations of each methodology. Moreover, the meta-analytic approaches carried out in this review have identified 35 different plant families and 60 edible sources, which can be used both as raw material for PAC extraction at industrial level and to introduce PACs through the diet. Finally, literature searches coupled with forest plot analyses have shown how PACs can have potential beneficial effects on human health. In particular, in this review we have explained how PACs can modulate the cholesterol content in the blood through a systematic action at different organ levels, or display local anti-inflammatory activity on the intestinal epithelium after the intake of PAC-enriched foods.

## Figures and Tables

**Table 1:** Documented plant physiological processes and plant responses to abiotic and biotic stresses that involve proanthocyanidins.

Condition		Effect on PAC Content	Plant Species	Ref.
No Stress	Germination	▲	*Phaseolus vulgaris*	[35]
			*Cucumis sativus*	[46,47]
		▽	*Sapium sebiferum*	[48]
			*Arabidopsis thaliana*	[49,50]
	Aging	▲	*Cistus clusii*	[51]
	Maturation	▽	*Fragaria ananassa*	[52,53]
Abiotic Stress	Excess Light	▲	*Malus domestica*	[54]
			*Larix gmelinii*	[55]
			*Cistus clusii*	[51]
			*Populus tremula*	[56,57]
	Heat Stress	▽	*Vitis vinifera*	[58,59]
		▲	*Cucumis sativus*	[47]
	Cold Stress	▲	*Fagopyrum tataricum*	[60]
			*Malus domestica*	[54,61,62]
			*Cucumis sativus*	[63,64]
	Water Deficit	▲	*Vitis vinifera*	[65,66,67]
	High Salinity	▲	*Arabidopsis thaliana*	[68]
			*Calliergon giganteum*	[69]
		▽	*Fagopyrum tataricum*	[70]
Biotic Stress	*Melampsora larici-populina*	▲	*Populus tremula*	[71,72]
	*Botrytis cinerea*		*Vitis vinifera*	[73,74]
	*Botrytis cinerea*		*Vaccinium myrtillus*	[75]
	*Botrytis cinerea*		*Fragaria ananassa*	[76]
	*Paraphaeosphaeria michotii*		*Vaccinium myrtillus*	[75]
	*Marssonina brunnea*		*Populus tremula*	[77]
	*Colletotrichum acutatum*		*Vitis vinifera*	[78]
	*Lymantria dispar*	▲	*Populus tremula*	[79]
	*Malacosoma disstria*		*Populus tremula*	[80]
	*Leucoma salicis*		*Populus tremula*	[80]

PACs: proanthocyanidins; ▲: increase of PAC content; ▽: decrease of PAC content.

**Table 2:** In vitro and in vivo studies on PAC-mediated glucose-lowering effect.

Glucose-Lowering/Anti-Diabetic Studies				
Reference		PACs Type or Source	Plasma Parameters	Model
Han et al., 2018	[185]	procyanidin B2	GLU	Mice
Yokozawa et al., 2012	[186]	PACs	GLU, GP, BUN	Rats
Hollands et al., 2018	[187]	EC and oligomeric PAC from apple	GLU, INS, fructosamine, TG, TC, HDL, LDL	Human
El-Alfy et al., 2005	[188]	grape seed	GLU, INS	Rats
Ding et al., 2013	[189]	grape seed	GLU, INS	Rats
Li et al., 2020	[190]	grape seed	GLU, BUN, DAO	Piglets
Pinent et al., 2004	[191]	GSPE	GLU, INS	Rats
Castell-Auvì et al., 2012	[192]	GSPE	INS	Rats
Bao et al., 2014	[193]	GSPE	GLU, albumin	Rats
Li et al., 2015	[194]	GSPE	GLU, INS, HbA1c	Rats
Chen et al., 2015	[195]	GSPE	GLU	Rats
Zhang et al., 2016	[196]	GSPE	GLU, INS, TG, TC	Rats
Sanna et al., 2019	[197]	GSPE	GLU, INS	Rats
Ding et al., 2020	[198]	GSPE	GLU, creatinine, BUN, uric acid, urinary albumin, renal MDA	Rats
Desideri et al., 2012	[179]	cocoa	GLU, INS, HOMA-IR, TC, TG, LDL, HDL, HDL	Human
Mellor et al., 2013	[199]	chocolate	GLU, INS, HbA1c, CRP, TC, TG, LDL, HDL	Human
Yamashita et al., 2019	[200]	cacao liquor	GLU, INS, GLP-1	Mice
Tomaru et al., 2007	[201]	cacao liquor	GLU, fructosamine	Mice
Yamashita et al., 2012	[202]	cacao liquor	GLU, INS	Mice
Rodríguez-Daza et al., 2020	[203]	blueberry	GLU, INS	Mice
Ntemiri et al., 2020	[204]	blueberry	GLU, CRP, FRAP	Human
Liu et al., 2020	[205]	white bayberry	GLU, INS, leptin, glucagon, TG, TC, LDL, ALT	Mice
Castro-Acosta et al., 2017	[206]	apple and blackcurrant	GLU, INS, CRP, GIP	Human
Kanamoto et al., 2011	[207]	black soybean seed	GLU, INS, HOMA-IR, TG, TC, leptin, adiponectin, NEFA	Mice
Lee et al., 2008	[208]	persimmon peel	GLU, GP, TC, TG, NEFA, OS biomarkers	Mice
Lin et al., 2018	[209]	cinnamon twig	GLU, TG, LDL-C, HDL-C, ALT, adiponectin, leptin	Rats
Hsu et al., 2020	[210]	*C. obtusa* var. formosana leaf	GLU, INS, leptin, AST, ALT, TG, TC, HDL, LDL, amylase, lipase	Rats
Macho-Gonzàlez et al., 2020	[211]	carob fruit extract	GLU, INS, HOMA-β index	Rats
Anunciação et al., 2018	[212]	extruded sorghum	GLU	Human
Wang et al., 2020	[213]	*C. osmophloeum* and *T. camphoratus*	GLU, TC, BUN, creatinine	Mice
Bang et al., 2014	[214]	Enzogenol	GLU, INS, HbA1c, glucagon	Mice

## Supplementary Materials

The following are available online at https://www.mdpi.com/article/10.3390/antiox10081229/s1, Figure S1: Funnel plot representation of the effects derived from the supplementation of PACs on hematic levels of sugar (A), cholesterol (B), HDL (C) and LDL (D).
