# Supplementary material for: Proanthocyanidins and Where to Find Them: A Meta-Analytic Approach to Investigate Their Chemistry, Biosynthesis, Distribution, and Effect on Human Health
Source: Antioxidants (Basel). 2021 Jul 30;10(8):1229. doi: 10.3390/antiox10081229 (PMC8389005; doi:10.3390/antiox10081229)
Supplement: Supplementary file 1 [file antioxidants-10-01229-s001.zip › antioxidants-1293729-supplementary.pdf]

Supplementary Materials

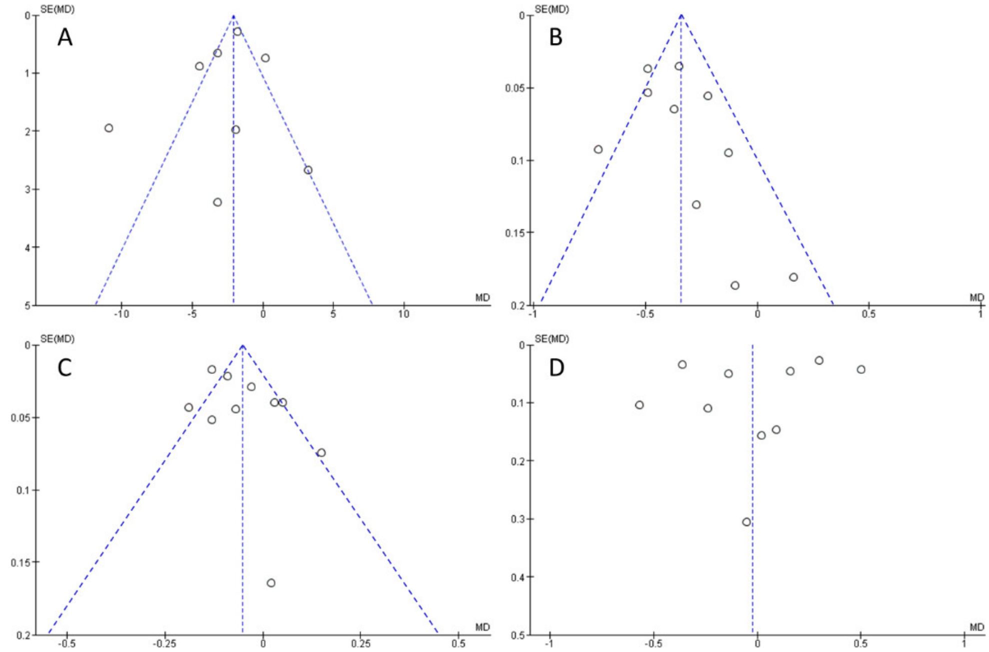

Figure S1: Funnel plot representation of the effects derived from the supplementation of PACs on hematic levels of sugar (**A**), cholesterol (**B**), HDL (**C**), and LDL (**D**). The x-axis shows the results for the selected studies plotted as the odds ratio. Each dot represents a single study. The figure was generated by Review Manager Software, version 5.4.1.
